# PpCas9 from *Pasteurella pneumotropica* — a compact Type II-C Cas9 ortholog active in human cells

**DOI:** 10.1093/nar/gkaa998

**Published:** 2020-11-05

**Authors:** Iana Fedorova, Aleksandra Vasileva, Polina Selkova, Marina Abramova, Anatolii Arseniev, Georgii Pobegalov, Maksim Kazalov, Olga Musharova, Ignatiy Goryanin, Daria Artamonova, Tatyana Zyubko, Sergey Shmakov, Tatyana Artamonova, Mikhail Khodorkovskii, Konstantin Severinov

**Affiliations:** Skolkovo Institute of Science and Technology, Center of Life Sciences, Moscow, 121205, Russia; Center for Precision Genome Editing and Genetic Technologies for Biomedicine, Institute of Gene Biology, Russian Academy of Sciences, Moscow, 119334, Russia; Skolkovo Institute of Science and Technology, Center of Life Sciences, Moscow, 121205, Russia; Center for Precision Genome Editing and Genetic Technologies for Biomedicine, Institute of Gene Biology, Russian Academy of Sciences, Moscow, 119334, Russia; Peter the Great St. Petersburg Polytechnic University, Saint Petersburg, 195251, Russia; Skolkovo Institute of Science and Technology, Center of Life Sciences, Moscow, 121205, Russia; Center for Precision Genome Editing and Genetic Technologies for Biomedicine, Institute of Gene Biology, Russian Academy of Sciences, Moscow, 119334, Russia; Peter the Great St. Petersburg Polytechnic University, Saint Petersburg, 195251, Russia; Peter the Great St. Petersburg Polytechnic University, Saint Petersburg, 195251, Russia; Saint Petersburg State University, Saint Petersburg, 199034, Russia; Center for Precision Genome Editing and Genetic Technologies for Biomedicine, Institute of Gene Biology, Russian Academy of Sciences, Moscow, 119334, Russia; Peter the Great St. Petersburg Polytechnic University, Saint Petersburg, 195251, Russia; Peter the Great St. Petersburg Polytechnic University, Saint Petersburg, 195251, Russia; Peter the Great St. Petersburg Polytechnic University, Saint Petersburg, 195251, Russia; Saint Petersburg State University, Saint Petersburg, 199034, Russia; Skolkovo Institute of Science and Technology, Center of Life Sciences, Moscow, 121205, Russia; Institute of Molecular Genetics of National Research Center “Kurchatov Institute’’, Moscow, 123182, Russia; Skolkovo Institute of Science and Technology, Center of Life Sciences, Moscow, 121205, Russia; Skolkovo Institute of Science and Technology, Center of Life Sciences, Moscow, 121205, Russia; Peter the Great St. Petersburg Polytechnic University, Saint Petersburg, 195251, Russia; National Center for Biotechnology Information, National Library of Medicine, National Institutes of Health, Bethesda, MD 20894, USA; Peter the Great St. Petersburg Polytechnic University, Saint Petersburg, 195251, Russia; Peter the Great St. Petersburg Polytechnic University, Saint Petersburg, 195251, Russia; Saint Petersburg State University, Saint Petersburg, 199034, Russia; Institute of Molecular Genetics of National Research Center “Kurchatov Institute’’, Moscow, 123182, Russia

## Abstract

CRISPR-Cas defense systems opened up the field of genome editing due to the ease with which effector Cas nucleases can be programmed with guide RNAs to access desirable genomic sites. Type II-A SpCas9 from *Streptococcus pyogenes* was the first Cas9 nuclease used for genome editing and it remains the most popular enzyme of its class. Nevertheless, SpCas9 has some drawbacks including a relatively large size and restriction to targets flanked by an ‘NGG’ PAM sequence. The more compact Type II-C Cas9 orthologs can help to overcome the size limitation of SpCas9. Yet, only a few Type II-C nucleases were fully characterized to date. Here, we characterized two Cas9 II-C orthologs, DfCas9 from *Defluviimonas sp.20V17* and PpCas9 from *Pasteurella pneumotropica*. Both DfCas9 and PpCas9 cleave DNA *in vitro* and have novel PAM requirements. Unlike DfCas9, the PpCas9 nuclease is active in human cells. This small nuclease requires an ‘NNNNRTT’ PAM orthogonal to that of SpCas9 and thus potentially can broaden the range of Cas9 applications in biomedicine and biotechnology.

## INTRODUCTION

CRISPR-Cas are bacterial and archaeal immune systems that degrade invaders genomes using RNA-guided Cas nucleases (1–3). The CRISPR-Cas loci consist of CRISPR arrays and CRISPR-associated *cas* genes (4). CRISPR arrays are composed of repeats separated by intervening unique spacers. Some spacers are derived from invaders DNA and are acquired by CRISPR arrays from genetic mobile elements such as plasmids or bacteriophages (5–7). The CRISPR array is transcribed into a long precursor crRNA and further processed to mature short CRISPR RNAs (crRNAs), each containing a part of repeat and a single spacer sequence (8,9). Mature crRNAs bind to Cas effector proteins and guide them to regions of invader genomes complementary to crRNA spacer segment (10). The specific recognition of nucleic acid targets leads to activation of Cas effector nucleases and subsequent degradation of invader's genome (1,2,9).

The ability to guide Cas nucleases to DNA targets of choice using crRNAs of different sequences led to development of efficient and easy-to-use genome engineering tools (11–13). CRISPR-Cas systems vary in terms of effector complexes architecture and mechanisms of action. In CRISPR-Cas class II systems’ effectors Cas9, the crRNA binding and DNA cleavage functions are combined in a single, albeit large proteins and this simplicity led to their extensive use (14).

The Cas9 effector nuclease of Type II-A CRISPR-Cas system from *Streptococcus pyogenes* was the first Cas nuclease to be successfully harnessed for genome engineering in human cells (13,15). Despite biochemical characterization of several other Type II-A Cas9 orthologs, the SpCas9 still remains the most investigated and highly used enzyme of its class due to its high efficiency and requirements for a relatively short PAM (protospacer adjacent motif)—several nucleotides flanking the target site that are essential for efficient DNA recognition and cleavage.

The Cas9 effector proteins of Type II-C CRISPR-Cas systems have generally a smaller size than Type II-A counterparts, which allows the simultaneous delivery of Type II-C Cas9 gene and sequences coding for guide RNAs in a single adeno-associated viral (AAV) particle (16–19). Type II-C effectors from *Neisseria meningitidis* strain 8013 (NmeCas9, (20)) and strain De11444 (Nme2Cas9, (18)), *Campylobacter jejuni* (CjCas9, (16)), *Corynebacterium diphtheriae* (CdCas9, (21)) and *Geobacillus stearothermophilus* (GeoCas9, (22)) were characterized and shown to mediate genome editing in human cells. Together with the small-sized Type II-A Cas9s from *Staphylococcus aureus* (SaCas9, (23)) and *Staphylococcus auricularis* (SauriCas9, (17)), these nucleases comprise a group of small Cas9 enzymes, whose use may be advantageous during the development of AAV-based genome editing platforms.

Despite the advantages of their size, small Cas9 nucleases characterized to date tend to require long PAMs—5′-NNGRRT-3′ for SaCas9; 5′-NNNVRYAC-3′ for CjCas9; 5′-NNNNGNTT-3′ for NmeCas9; 5′-NNRHHHY-3′ for CdCas9; 5′-NNNNCRAA-3′ for GeoCas9—which narrows the choice of targets available for editing. Protein engineering of these enzymes as well as further searches and characterization of new small-size nucleases with shorter PAMs helps to expand the tool kit of genomic editors and increase the number of editable sites (24). Indeed, two recently characterized small orthologs Nme2Cas9 and SauriCas9 require short PAM sequences (5′-NNNNCC-3′and 5′-NNGG-3′, correspondingly), indicating that the natural diversity of small-sized Cas9 editors can be harnessed to provide a viable alternative to and/or complement the currently widely used enzymes.

Here, we functionally characterized two small-sized Type II-C Cas9 orthologs from *Defluviimonas sp.20V17*, a bacterium inhabiting deep-sea hydrothermal vents (25,26) and *Pasteurella pneumotropica* (*Rodentibacter pneumotropicus*), a gram-negative bacterium isolated from multiple mammalian species (27). Using *in vitro* studies and/or experiments in bacteria we show that *Defluviimonas sp.20V17* and *P. pneumotropica* CRISPR-Cas Type II-C systems encode active Cas9 nucleases that efficiently cleave target DNA with novel, 5′-NNRNAY-3′ (DfCas9) and 5′-NNNNRT-3′ (PpCas9) PAMs. In contrast to DfCas9, the PpCas9 nuclease exhibits activity in human cells, introducing indels in HEK293T genome targets flanked by a 5′-NNNNRTT-3′ PAM.

## MATERIALS AND METHODS

### Plasmids cloning

The predicted CRISPR-Cas Type II-C system locus of *Defluviimonas sp.20V17* including three spacers in the CRISPR array was polymerase chain reaction (PCR) amplified with primers locus_DfCas9_F and locus_DfCas9_R using *Defluviimonas sp.20V17* genome DNA (DSMZ 24802) as a template. The resulting fragment was inserted into XbaI and HindIII digested pACYC184 vector using NEBuilder HiFi DNA Assembly Cloning Kit (NEB, E5520).

To obtain pET21a_DfCas9 plasmid, DfCas9 coding sequence was PCR amplified with DfCas9_F and DfCas9_R primers using bacterial genome DNA as a template. To obtain pET21a_PpCas9 plasmid, PpCas9 coding sequence was synthetized as g-block (IDT). The DfCas9 or PpCas9 coding fragments were inserted into XhoI and NheI digested pET21a vector by NEBuilder HiFi DNA Assembly Cloning Kit (NEB, E5520). The vectors maps and primers are presented in the Supplementary Table S1.

For expression in human cells PpCas9 gene was codon-optimized and inserted into plasmid under regulation of CMV promoter. SgRNA expression was driven by U6 promoter. The vector map is presented in the Supplementary Table S1.

### Plasmid transformation interference screening

To determine DfCas9 PAM sequence a randomized 7N plasmid library carried a protospacer sequence flanked by seven randomized nucleotides was used (Supplementary Table S1). To create the library the ssDNA oligo Library_F containing randomized nucleotides was double-stranded through single stage PCR with Library_R primer (Evrogen). This fragment was assembled with PUC19 fragment synthesized through PCR using primers PUC19_F and PUC19_R by NEBuilder HiFi DNA Assembly Cloning Kit (NEB, E5520). The mix was transformed to *Escherichia coli* DH5alpha strain and plated to media supplemented with 100 μg/ml ampicillin. The plates were incubated at 37°C. Eighteen hours after transformation more than 50 000 colonies were washed off the plates, and the plasmid library was extracted by Qiagen Plasmid Maxi kit (Qiagen 12162). The library plasmid map is presented in the Supplementary Table S1. Competent *E. coli* Star cells carrying pACYC184_DfCas9_locus or an empty pACYC184 vector were transformed with 7N PAM plasmid libraries and plated to 100 μg/ml ampicillin and 25 μg/ml chloramphenicol containing agar plates. After 16 h cells were harvested and DNA was extracted using Qiagen Plasmid Maxi kit (Qiagen 12162). PAM-coding sequences were PCR amplified using M13_f and M13_r primers and sequenced using Illumina platform with pair-end 150 cycles (75+75).

### Bacterial RNA sequencing

*Escherichia coli* DH5alpha carrying pACYC184_DfCas9_locus plasmid were grown for 16 h at 37°C in LB (Luria Bertani) medium supplemented with 25 μg/ml chloramphenicol. Bacteria were resuspended in TRIzol (Thermo Fisher Scientific, 15596026). Total RNA was purified using Direct-Zol RNA kit (Zymo research, R2051). RNA was DNase I (Zymo research) treated, 3′ dephosphorylated with T4 PNK (NEB, M0201) and next treated with RNA 5′ Polyphosphatase (Lucigen, RP8092H). Ribo-Zero rRNA Removal Kit (Gram-Negative Bacteria) kit (Illumina, 15066012) was used to remove ribosomal RNA. HTS samples were prepared using NEBNext Multiplex Small RNA Library Prep Set for Illumina (NEB, E7300). The library was sequenced using Illumina platform with pair-end 150 cycles (75+75).

### RNA sequencing analysis

HTS results of RNA sequencing were aligned to the reference plasmid pACYC184_DfCas9_locus using BWA aligner (28). Determined coordinates of 5′ and 3′ RNA ends were used to reconstruct the full-length RNA sequences. The resulting fragments were analyzed using Geneious 11.1.2. Filtered 40–130 nt-length sequences were used to generate the alignment.

### *In vitro* DNA cleavage assays

DNA cleavage reactions were performed using the recombinant DfCas9 or PpCas9 proteins and linear dsDNA targets. The reaction conditions were: 1 × CutSmart (NEB, B7204) buffer, 0.5 mM DTT (1,4-Dithiothreitol), 20 nM DNA, 400 nM recombinant protein, 2 μM crRNA, 2 μM tracrRNA.

Samples were incubated at 37°C (DfCas9) or 42°C (PpCas9) for 30 min (unless otherwise stated). Further, 4× loading dye containing 10 mM Tris–HCl, pH 7.8, 40% glycerol, 40 mM ethylenediaminetetraacetic acid, 0.01% bromophenol blue, 0.01% xylene cyanol was added to stop the reaction. Reaction products were analyzed by electrophoresis in 1.5% agarose gels. Pre-staining with ethidium bromide was used for visualization of bands on agarose.

For *in vitro* PpCas9 PAM screening, 100 nM linear DNA 7N PAM library was incubated with 400 nM recombinant protein, 5 μM crRNA, 5 μM tracrRNA. Reactions without crRNA were used as negative controls. The reaction was performed at 42°C for 30 min. Reaction products were separated by electrophoresis in agarose gels. Uncleaved DNA fragments were extracted from the gel using Zymo Clean Gel Recovery kit (Zymo research, D4007). HTS libraries were prepared using Ultra II DNA library prep kit (NEB, E7646). Samples were sequenced using MiniSeq Illumina with pair-end 300 cycles.

For testing the activity of DfCas9 or PpCas9 at different temperatures a mix of the corresponding protein with *in vitro* transcribed crRNA-tracrRNA in the cleavage buffer, and the DNA substrates, also in the cleavage buffer, were first incubated separately at the chosen temperature for 2 min, combined and incubated for additional 10 min at same temperature. The following concentrations were used: 12 nM DNA, 240 nM PpCas9 or DfCas9, 1,2 μM crRNA, 1,2 μM tracrRNA—for *in vitro* cleavage of a linear DNA fragment; 4 nM DNA, 80 nM PpCas9 or DfCas9, 400 nM crRNA, 400 nM tracrRNA—for *in vitro* cleavage of a plasmid DNA.

All RNAs used in this study are listed in Supplementary Table S2.

### Computational sequence analysis

For analysis of PpCas9 *in vitro* PAM screening results as well as DfCas9 plasmid interference screening in bacteria, Illumina reads were filtered by requiring an average Phred quality (Q score) of at least 20. Resulting reads were mapped against the corresponding reference sequence using BWA (28). All unmapped reads were discarded from the analysis. The degenerate 7-nt region was extracted from the sequences. A total 16 301 unique PAM sequences were found both for the depleted and control samples for DfCas9 PAM screening, and 16 384 unique PAM sequences were found for PpCas9 PAM screening analysis. The median coverage of every individual PAM variant comprised 120 and 790 reads for DfCas9 and PpCas9 PAM screening, respectively. WebLogo was used to generate logo based on statistically significantly (one-sided Pearson chi-square test with a *P*-value less than 10^−12^) depleted PAM sequences (122 and 79 PAMs for DfCas9 and PpCas9, respectively; Supplementary Files S2 and 3). For PAM wheel construction the depletion values of three nucleotide sequences (5–7 PAM positions) were counted similarly to Maxwell *et al.* (29): Dсoef = log2[[total N sample/ total N control] *[N PAM control/N PAM sample]], where N is a number of reads. PAM sequences with positive depletion coefficient were used as an input for PAM wheel construction.

### Recombinant protein purification

For recombinant DfCas9 and PpCas9 purification competent *E. coli* Rosetta cells were transformed with pET21a_DfCas9 or pET21a_PpCas9 plasmid and grown till OD_600_ = 0.6 in 500 ml LB media supplemented with 100 μg/ml ampicillin. The target protein synthesis was induced by the addition of 1 mM IPTG. After 5 h of growth at 25°C, cells were centrifugated at 4000 g, the pellet was resuspended in lysis buffer containing 50 mM Tris–HCl pH 8.0 (4°C), 500 mM NaCl, 1 mM beta-mercaptoethanol and 10 mM imidazole supplemented with 1 mg/ml lysozyme (Sigma) and cells were lysed by sonication. The cell lysate was centrifuged at 16000g (4°C) and filtered through 0.45 μm filters. The lysate was applied to 1 ml HisTrap HP column (GE Healthcare) and DfCas9 or PpCas9 was eluted by 300 mM imidazole in the same buffer without lysozyme. After affinity chromatography fractions containing the nuclease were applied on a Superdex200 Increase 10/300 GL (GE Healthcare) column equilibrated with a buffer containing 50 mM Tris–HCl pH 8.0 (4°C), 500 mM NaCl, 1 mM DTT (1,4-Dithiothreitol). Fractions containing DfCas9 or PpCas9 monomer were pooled and concentrated using 30 kDa Amicon Ultra-4 centrifugal unit (Merc Millipore, UFC803008). Glycerol was added to final concentration of 10% and samples were flash-frozen in liquid nitrogen and stored at −80°C. Purity of the nucleases was assessed by denaturing 8% polyacrylamide gel electrophoresis and the integrity of recombinant protein was confirmed by mass spectrometry.

### Cell culture and transfection

HEK293RT cells were maintained in Dulbecco's modified Eagle's Medium supplemented with 10% fetal bovine serum at 37°C with 5% CO_2_ incubation. Cells were seeded into 24-well plates (Eppendorf) one day prior to transfection. Cells were transfected using Lipofectamine 2000 (Thermo Fisher Scientific) following the manufacturer's recommended protocol. For each well of a 24-well plate a total of 500 ng plasmids was used. Three days after transfection cells were harvested and genomic DNA was extracted using QuickExtract solution (Lucigen, QE0950).

### Indel frequency analysis

The genomic DNA from transfected cells was obtained as described above. Genomic region surrounding the CRISPR target site was amplified using two-step PCR. At the first step primers combining target-specific sequences and Illumina adapter overhangs were used (Supplementary Table S5).

First-Round PCR Forward Primer:

5′ **CTCTTTCCCTACACGACGCTCTTCCGATCT**NNNN [target-specific sequence] 3′

First-Round PCR Reverse Primer:

5′ **TCAGACGTGTGCTCTTCCGATCT** [target-specific sequence] 3′

The result amplicons were used as template in the second step PCR. This step introduced 8N barcode and flow cell linker adaptors using primers containing a sequence that anneals to the Illumina primer sequence introduced in first step.

Second-Round PCR Forward Primer:

5′AATGATACGGCGACCACCGAGATCTACA**CTCTTTCCCTACACGACGCTCTTCCGATCT** 3′

Second-Round PCR Reverse Primer:

5′CAAGCAGAAGACGGCATACGAGAT**NNNNNNNN**GTGACTGGAGT**TCAGACGTGTGCTCTTCCGATCT** 3′

The second round PCR products were loaded to agarose electrophoresis. The PCR fragments were gel-extracted using Cleanup Standard kit (Evrogen, BC022) and sequenced using Illumina (pair-end 150 + 150 or 75 + 75 cycles). Illumina reads were checked for the number of substitutions in regions covering sequences of primers used in PCR. These regions by experiment design don’t include any indels and shouldn’t contain a lot of errors. Thus, this step allows to filter out erroneous reads. As a threshold we used an average error rate 0.24 ± 0.06% per base proposed by Pfeiffer *et al.* (30) Filtered reads were merged using custom script. Indel frequencies were estimated using CRISPResso2 analysis package (29). The window of 20 bp around the gRNA site and quantification window center corresponding to 3 nt from the 3′ end of the guide were provided to detect possible mutations. The resulting indel percentage was calculated as [Indels % in transfected cells]—[Indels % in untransfected cells] for a certain region of genomic DNA. Mann-Whitney U test was used to test the significance of the difference between the indel percentage in transfected and untransfected cells (negative control).

For T7 endonuclease I indel detection assay genomic region surrounding the CRISPR target site was PCR amplified using primers listed in Supplementary Table S1. The PCR fragments were gel-extracted using Cleanup Standart kit (Evrogen, BC022). Next, the indel detection assay was performed using T7 endonuclease I (NEB, M0302) according to manufacturer's recommended protocol. In brief, after incubation with T7 endonuclease I PCR products were loaded to agarose native gel and stained with ethidium bromide for 10 min.

## RESULTS

### *Defluviimonas sp.20V17* and *P. pneumotropica* CRISPR Cas II-C systems: organization of the loci

Bioinformatics searches of small-sized Cas9 proteins reveal multiple orthologues belonging to Type II-C type CRISPR-Cas systems (31–33). Most of these proteins are not biochemically characterized. CRISPR-Cas systems from *Defluviimonas sp.20V17* and *P. pneumotropica* carry intact sequences of *cas* genes and were chosen for further characterization.

**Figure 1.**
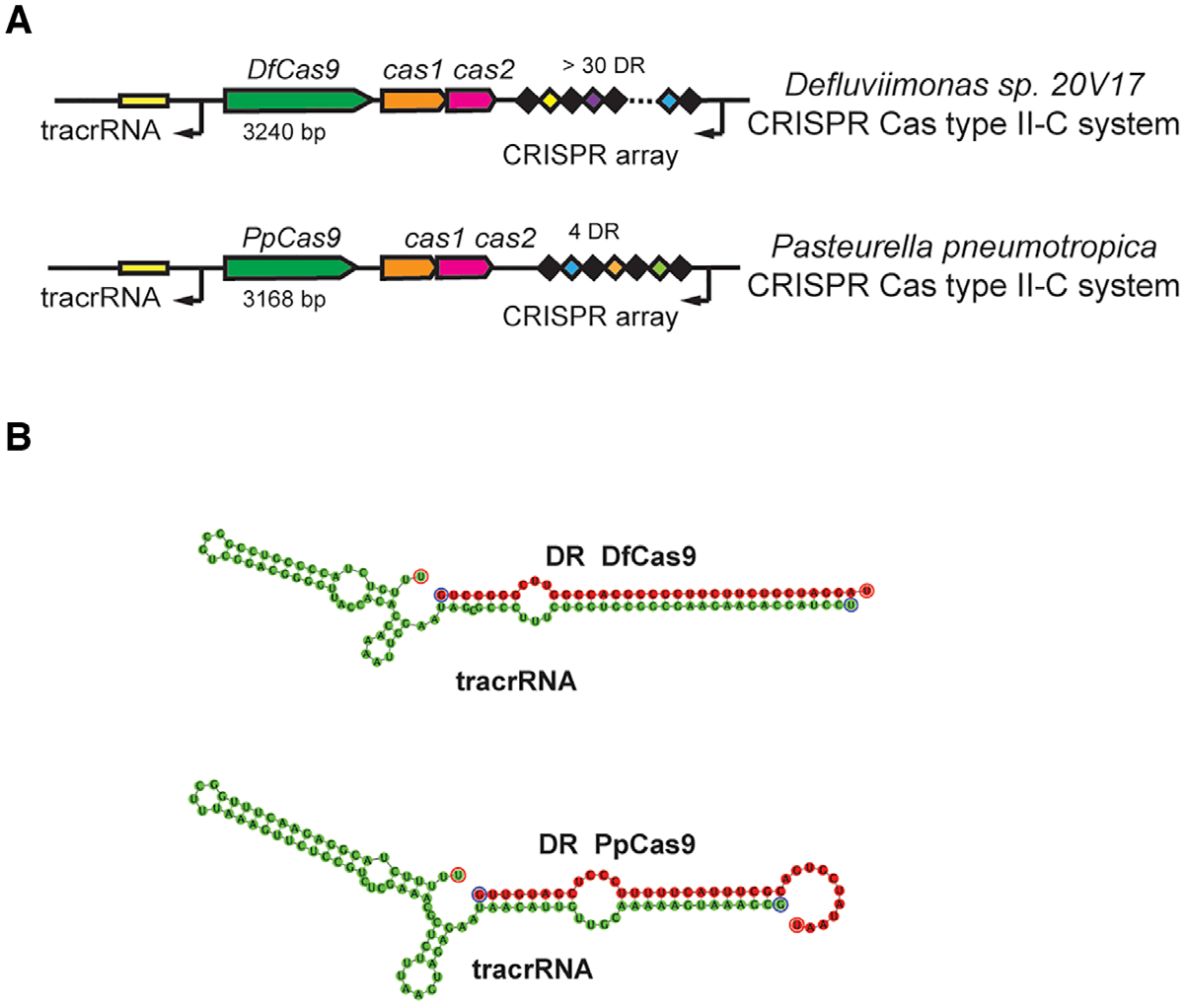
*Defluviimonas sp. 20V17* and *Pasteurella pneumotropica* CRISPR-Cas Type II-C loci. (**A**) Organization of *Defluviimonas sp. 20V17* and *P. pneumotropica* CRISPR-Cas Type II-C loci. DRs are shown as black rectangles, spacers are indicated by rectangles of different colors. The tracrRNA coding sequences are shown as yellow rectangles. The *cas* genes are labeled. Direction of transcription is indicated with black arrows. (**B**) *In silico* co-folding of *Defluviimonas sp. 20V17 and P. pneumotropica* DRs and putative tracrRNAs. The DR sequences are colored in red, the tracrRNA sequences are colored in green.

The *P. pneumotropica* CRISPR-Cas Type II-C locus contains an array composed of four 36-bp DRs (direct repeats) interspaced by 30-bp spacers in the proximity of the *cas* genes operon (Figure 1A). The available *Defluviimonas sp.20V17* genome assembly is fragmented and comprises 236 discrete contigs (26). The *Defluviimonas sp.20V17* Type II-C CRISPR-Cas system is located at the end of one contig and the leader-proximal part of the array is missing. Based on available information, the *Defluviimonas sp.20V17* Type II-C CRISPR array contains at least 30 DRs interspaced by 30-bp spacers (Figure 1A). A BLAST search using spacer sequences from arrays of both systems as queries revealed no matches to sequences from public databases. The adaptation modules of *P. pneumotropica* and *Defluviimonas sp.20V17* Type II-C CRISPR-Cas loci include the *cas1* and *cas2* genes. Each locus contains a *cas9* gene encoding relatively small Type II-C effectors: DfCas9 is 1079 amino acids long while PpCas9 is 1055 amino acids long. Upstream of *cas* genes in both systems we identified a putative tracrRNA-encoding sequences partially complementary to DRs. In both cases *in silico* co-folding of part of DR with the putative tracrRNA predicts stable secondary structures (Figure 1B).

### Characterization of *Defluviimonas sp.20V17* DfCas9 nuclease

**Figure 2.**
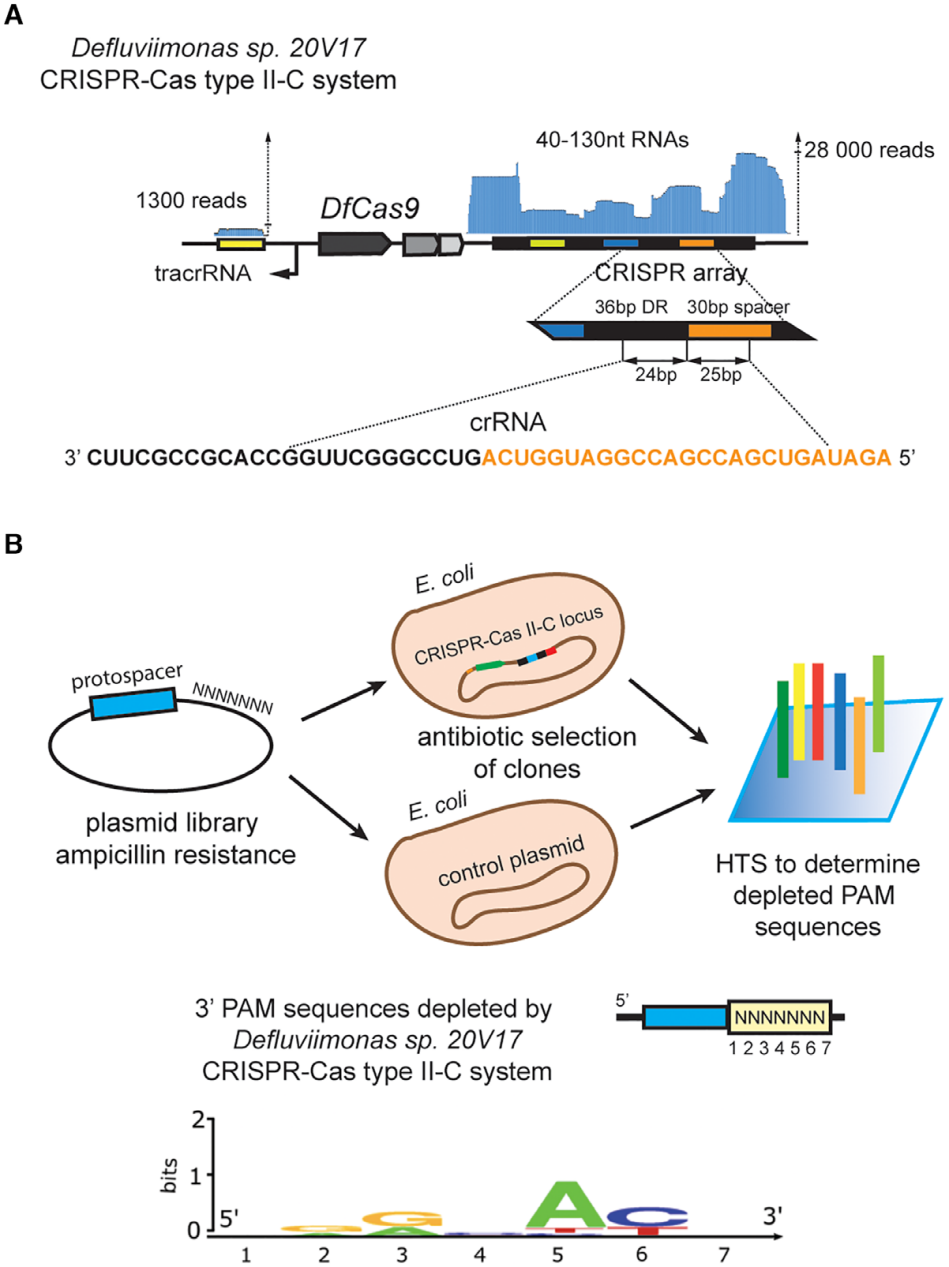
Studying of *Defluviimonas sp. 20V17* CRISPR-Cas Type II-C system in bacteria. (**A**) Identification of *Defluviimonas sp. 20V17* crRNAs. Reads (blue) are mapped at the top of the CRISPR array. A sequence of a typical mature crRNA sequence is expanded at the bottom with spacer part shown in orange. The direction of transcription is indicated with black arrows. (**B**) Determination of DfCas9 PAM sequences using plasmid transformation interference screening. Above: a scheme of the interference screen experiment. Below: DfCas9 PAM sequence logo determined from the PAM screening. PAM position numbers correspond to nucleotides immediately following the protospacer in the 5′-3′ direction.

To study *Defluviimonas sp.20V17* CRISPR-Cas Type II-C system we cloned the corresponding locus into the pACYC184 plasmid for heterologous expression. Only a fragment of CRISPR array containing four DRs adjacent to *cas* genes was used for cloning. To check the efficiency of transcription of RNA components of the cloned CRISPR-Cas system, small RNAs present in *E. coli* harboring plasmid-borne *Defluviimonas sp.20V17* locus were sequenced. We found that the shortened *Defluviimonas sp.20V17* CRISPR array was actively transcribed in an orientation opposite to that of *cas* genes transcription and processed crRNAs with 24–25 nt spacer-derived and 24 nt DR-derived segments were detected (Figure 2A). The tracrRNAs coding sequence was also expressed generating 72–83 nt products.

Given high expression levels of *Defluviimonas sp. 20V17* crRNA and tracrRNA in *E. coli*, we performed plasmid transformation interference screening in the heterologous host to determine the DfCas9 protospacer adjacent motif (PAM) sequence (Figure 2B). The plasmid transformation interference screening is based on transformation of *E. coli* cells carrying a plasmid with a CRISPR-Cas locus or an empty vector with a library of compatible plasmids bearing a protospacer sequence matching one of the spacers in the CRISPR array and flanked by seven randomized nucleotides. Transformed cells are plated on a medium that selects for cells carrying both plasmids. Since successful recognition of targets with interference-proficient PAM by Cas9 nuclease leads to plasmid destruction, under-representation of interference-proficient PAM library members is expected in transformants carrying the CRISPR-Cas locus comparing to control cells.

One of the spacers in *Defluviimonas sp. 20V17* CRISPR array was used as a protospacer for plasmid PAM library construction. Plasmid transformation interference screening in *E. coli* and subsequent high-throughput sequencing of the targeted protospacer region amplified from plasmids extracted from pooled transformant colonies revealed depletion of library members with 5′-NNRNAYN-3′ sequences adjacent to protospacer in cells carrying *Defluviimonas sp. 20V17* CRISPR-Cas Type II-C locus compared to control cells.

**Figure 3.**
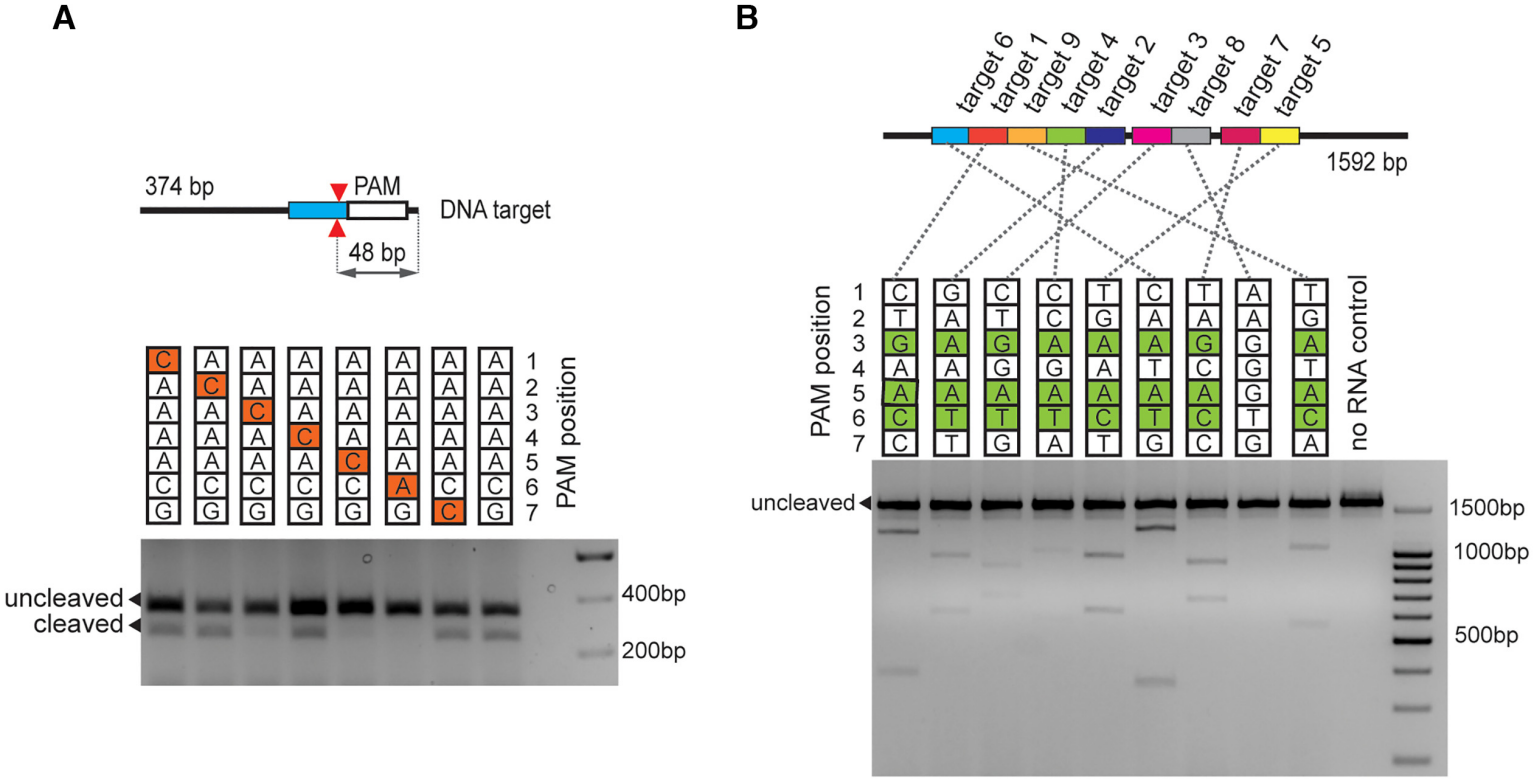
*In vitro* cleavage of DNA targets by DfCas9. (**A**) Single-nucleotide substitutions in the third, fourth and sixth positions of PAM prevent DNA cleavage by DfCas9. An agarose gel showing the results of electrophoretic separation of cleavage products of targets with PAM sequences shown at the top is presented. Bands corresponding to cleaved and uncleaved DNA fragments are indicated. The scheme above shows the position of expected DNA cleavage site. (**B**) DfCas9 cleaves different DNA targets with 5′-NNRNAYN-3′ PAM consensus *in vitro*. The scheme above shows the positions of different target sites in the grin2b gene fragment. Below: A gel showing results of *in vitro* cleavage of targets with indicated PAMs is presented.

To reconstruct the DfCas9 DNA cleavage reaction *in vitro*, recombinant DfCas9 was purified and crRNA and tracrRNA molecules were synthesized by T7 RNA polymerase (Supplementary File S1, Figure S1 and Table S3). As a DNA target we used a linear DNA fragment carrying a protospacer sequence flanked by 5′-AAAAACG-3′ PAM selected based on plasmid transformation interference screening results. The incubation of DfCas9–crRNA–tracrRNA ribonucleoprotein complex with the DNA target in a buffer supplemented with Mg^2+^ at 37°C for 30 min led to DNA cleavage (Figure 3A). To further examine the DfCas9 PAM preferences, single-nucleotide substitutions in the deduced consensus PAM sequence were introduced and individually tested for cleavage efficiency (Figure 3A). The replacement of purines to pyrimidines in the third position, as well as substitutions in the fifth and sixth positions of the 5′-AAAAACG-3′ PAM prevented DNA cleavage. These results confirmed the PAM consensus determined by plasmid transformation interference screening and allowed us to conclude that DfCas9 nuclease requires a 5′-NNRNAY-3′ PAM.

Next, we tested DfCas9 DNA cleavage activity on different targets. Several 20-bp targets flanked by 5′-NNRNAYN-3′ consensus PAM sequence were chosen on a 1592 bp linear DNA fragment of the GRIN2b gene. DNA cleavage reactions were performed using crRNAs DfCas9 charged with crRNAs corresponding to different target sites (Figure 3B and Supplementary Table S3). As can be seen, DfCas9 nuclease successfully introduced double-stranded breaks in all DNA targets, and did not cleave a site with an 5′-AAGGGTG-3′ located at the place of PAM, which was used as a negative control.

Overall, we conclude that DfCas9 nuclease specifically cleaves DNA targets flanked with 5′-NNRNAY-3′ PAM sequence at the 3′ side of protospacers.

### Characterization of PpCas9 nuclease from *Pasteurella pneumotropica*

**Figure 4.**
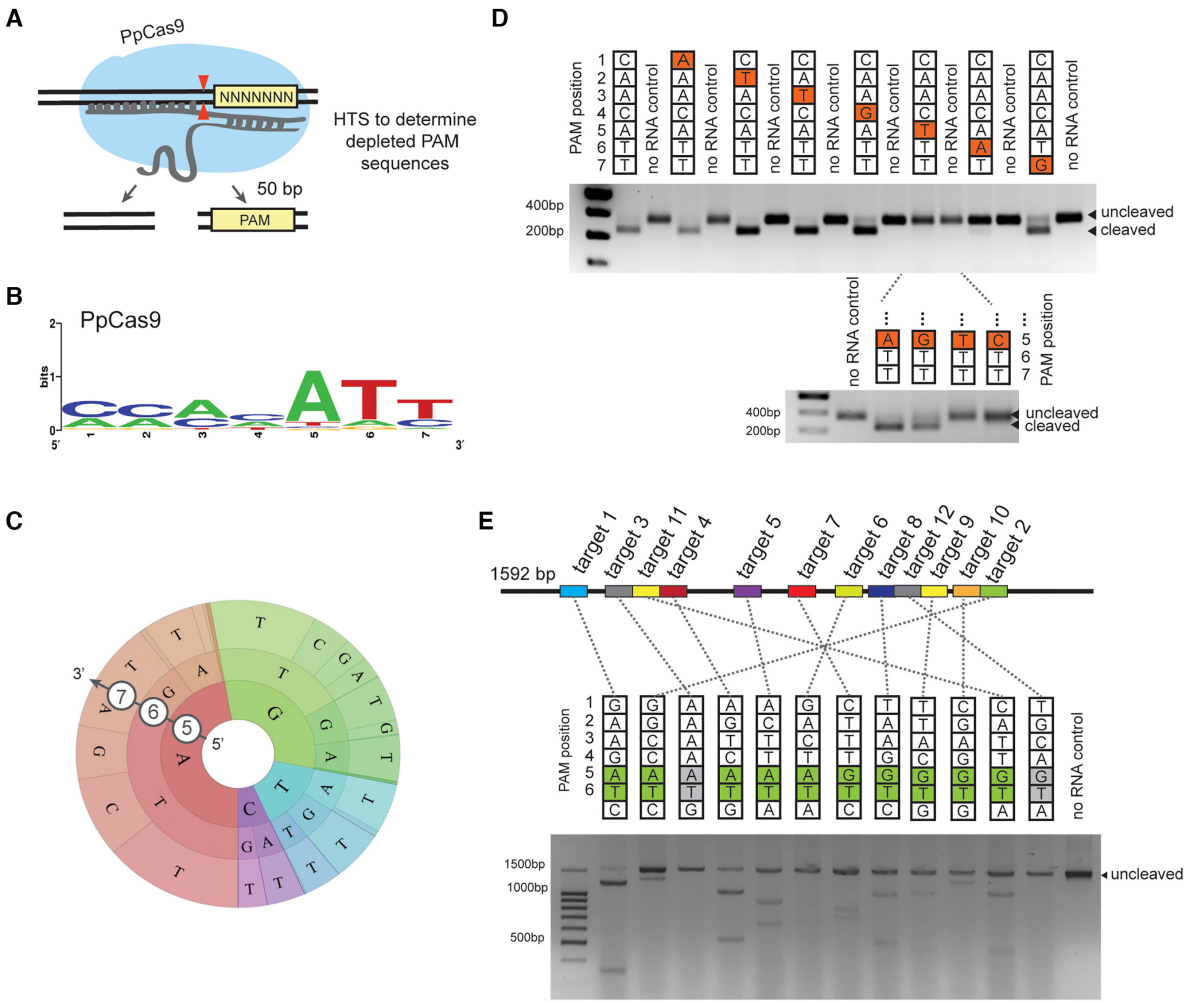
*In vitro* cleavage of DNA targets by PpCas9. (**A**) A scheme of the *in vitro* PAM screening experiment. A linear DNA PAM library containing a target site flanked with seven randomized nucleotides at the 3′ end is incubated with PpCas9 charged with appropriate crRNA and tracrRNA. This leads to the cleavage of library members carrying functional PAM sequences and generates DNA products shortened by 50 bp. Uncleaved PAM library molecules are recovered, which depletes the library. The uncleaved molecules, as well as negative control (original PAM library incubated with PpCas9-tracrRNA in the absence of crRNA) are sequenced. Comparison of PAM variants representation in the depleted sample and the control allows to determine the PpCas9 PAM logo. (**B**) Web logo of PpCas9 PAM sequences depleted after *in vitro* PAM screening. (**C**) Wheel representation of *in vitro* PAM screen results for fifth, sixth and seventh nucleotide positions of PAM. Nucleotide positions from the inner to the outer circle match PAM positions moving away from the protospacer. For a given sequence, the area of the sector in the PAM wheel displays the relative depletion in the library. (**D**) Single-nucleotide substitutions in the fifth and sixth positions of PAM prevent DNA cleavage by PpCas9. An agarose gel showing the results of electrophoretic separation of targets with PAM sequences shown at the top after incubation during 30 min with the PpCas9 effector complex is presented. Bands corresponding to cleaved and uncleaved DNA fragments are indicated. (**E**) PpCas9 efficiently cleaves different DNA targets with 5′-NNNNRTN-3′ PAM consensus *in vitro*. The scheme above shows the positions of different target sites in a 1592 bp GRIN2b gene fragment. Below: A gel showing results of *in vitro* cleavage of targets with indicated PAMs is presented.

Due to the lack of *P. pneumotropica* genomic DNA at our disposal, all experiments with the PpCas9 effector nuclease were performed *in vitro*. A recombinant PpCas9 was purified from *E. coli* Rosetta cells, the bioinformatically predicted crRNAs and tracrRNA were synthesized *in vitro*. To assess the PpCas9 nuclease activity we tested its ability to cleave linear DNA PAM libraries containing a target site flanked with seven randomized nucleotides at the 3′ end (Figure 4A). PpCas9 in complex with crRNA and tracrRNA was incubated with PAM library at 42°C for 30 min, uncleaved molecules were purified after agarose gel electrophoresis and sequenced (along with negative control—original PAM library incubated with PpCas9-tracrRNA in the absence of crRNA) using an Illumina platform. Comparison of PAM variants representation in the depleted sample and the control allowed us to determine the PpCas9 PAM logo. The results showed that PpCas9 prefers targets flanked by a 5′-NNNNATN- 3′ PAM (Figure 4B).

To further investigate PpCas9 PAM sequence preferences, identify individual sequences representing functional PAMs, and determine the relative activity of each PAM sequence, we used the PAM wheel approach for results visualization (34). PAM wheel is a Krona plot, a hierarchical node-link diagram, which allows one to detect the interrelationships between nucleotides in different PAM positions. The PAM wheel confirmed the importance of T in the sixth position and variability of the seventh PAM nucleotide with a slight bias for T. In addition, this approach revealed a preference for purines in the fifth position (Figure 4C).

Next, we made single-nucleotide substitutions in consensus 5′-CAACATT-3′ PAM and tested targets harboring PAM variants individually for PpCas9 cleavage efficiency (Figure 4D and Supplementary Figure S2). The results confirmed that there is no strict sequence-specific requirement to first four nucleotides of PAM; the preference for either a G or an A in the fifth position, and the importance of T in the sixth position. In addition, using similar experiments we confirmed that PpCas9, while tolerant for all four possible nucleotides substitutions in the seventh position of PAM, has a slight preference to T (Supplementary Figure S2). No preference for any nucleotides was detected in the eighth, ninth or tenth positions (Supplementary Figure S2).

Overall, the results allowed us to disregard the small preference for a T in the seventh position and conclude that for efficient *in vitro* DNA cleavage PpCas9 requires a 5′-NNNNRTN-3′ consensus PAM. To validate the proposed PAM consensus we tested whether PpCas9 is able to cleave different DNA targets flanked with this consensus. A 1592 bp linear DNA fragment was used as a target bearing several 20-nt PpCas9 target sites flanked by 5′-NNNNRTN-3′ PAM (Figure 4E). PpCas9 successfully cleaved most targets, confirming the deduced PAM consensus.

### DfCas9 and PpCas9 temperature preferences

**Figure 5.**
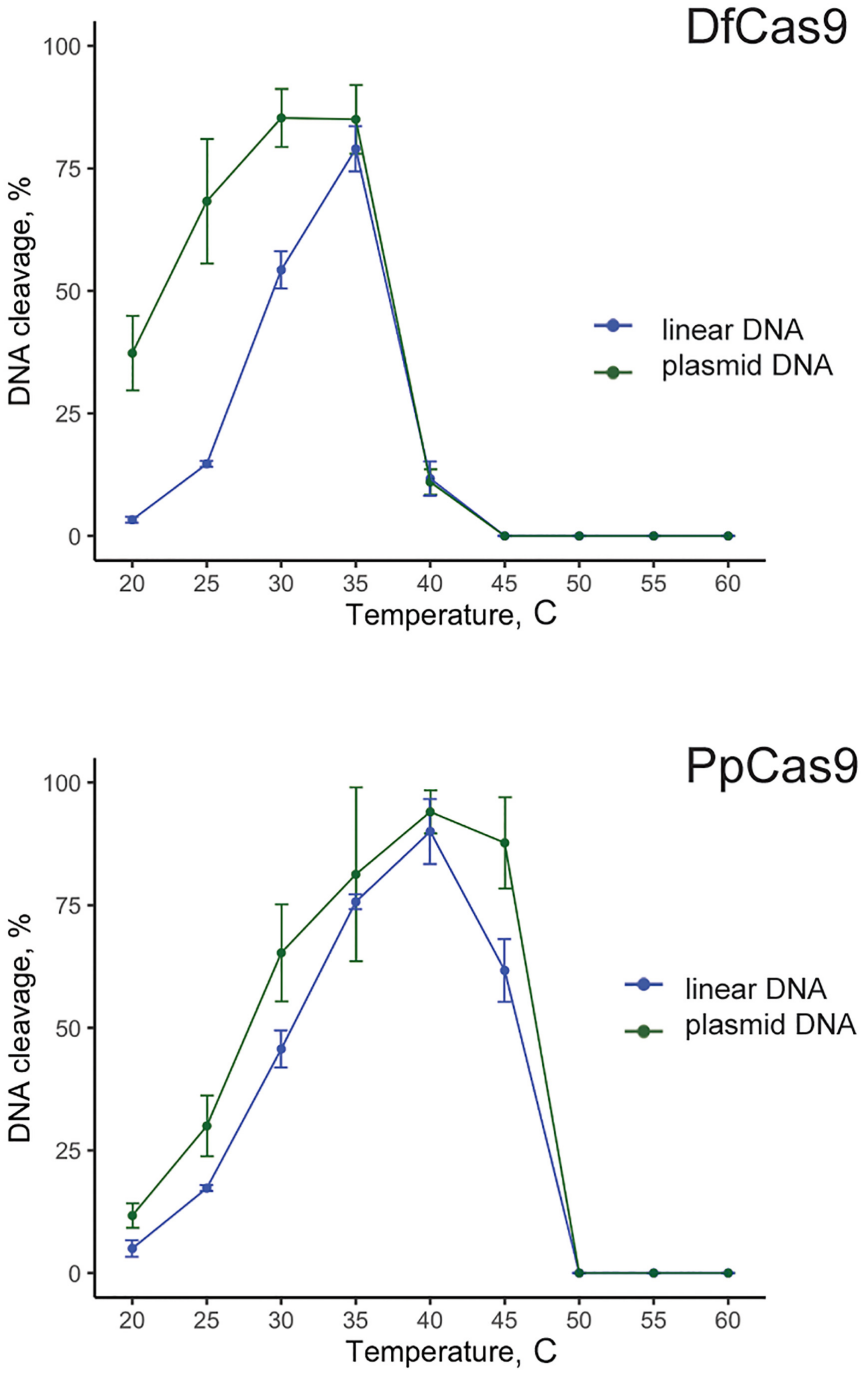
DNA cleavage activity of DfCas9 and PpCas9 at different temperatures. DfCas9 or PpCas9 was incubated with cognate tracrRNA and crRNA and a 2.7 kb plasmid DNA or a 921 bp linear DNA fragment containing target sequences at indicated temperatures for 10 min. Products were separated by agarose gel electrophoresis. Cleavage efficiency (in percent) was calculated as a ratio of intensity of cleaved bands to the combined intensity of cleaved and uncleaved bands. Mean values and standard deviations obtained from three independent experiments are shown.

The range of optimal working temperatures is one of the factors which determine Cas nuclease application. Temperature dependence of DfCas9 and PpCas9 nuclease activities was determined using targets flanked by corresponding consensus PAMs on either a linear DNA fragment or on a plasmid (Figure 5). DfCas9 efficiently cleaved the plasmid DNA in a temperature range of 20–37°C with maximal cleavage at 35°C. PpCas9 demonstrated lower activity at 20°C and efficiently cleaved its targets between 25 and 47°C with maximal activity at 40°C. In contrast to PpCas9, DfCas9 demonstrated different efficiencies of linear and supercoiled DNA cleavage.

### DfCas9 and PpCas9 sgRNA design

**Figure 6.**
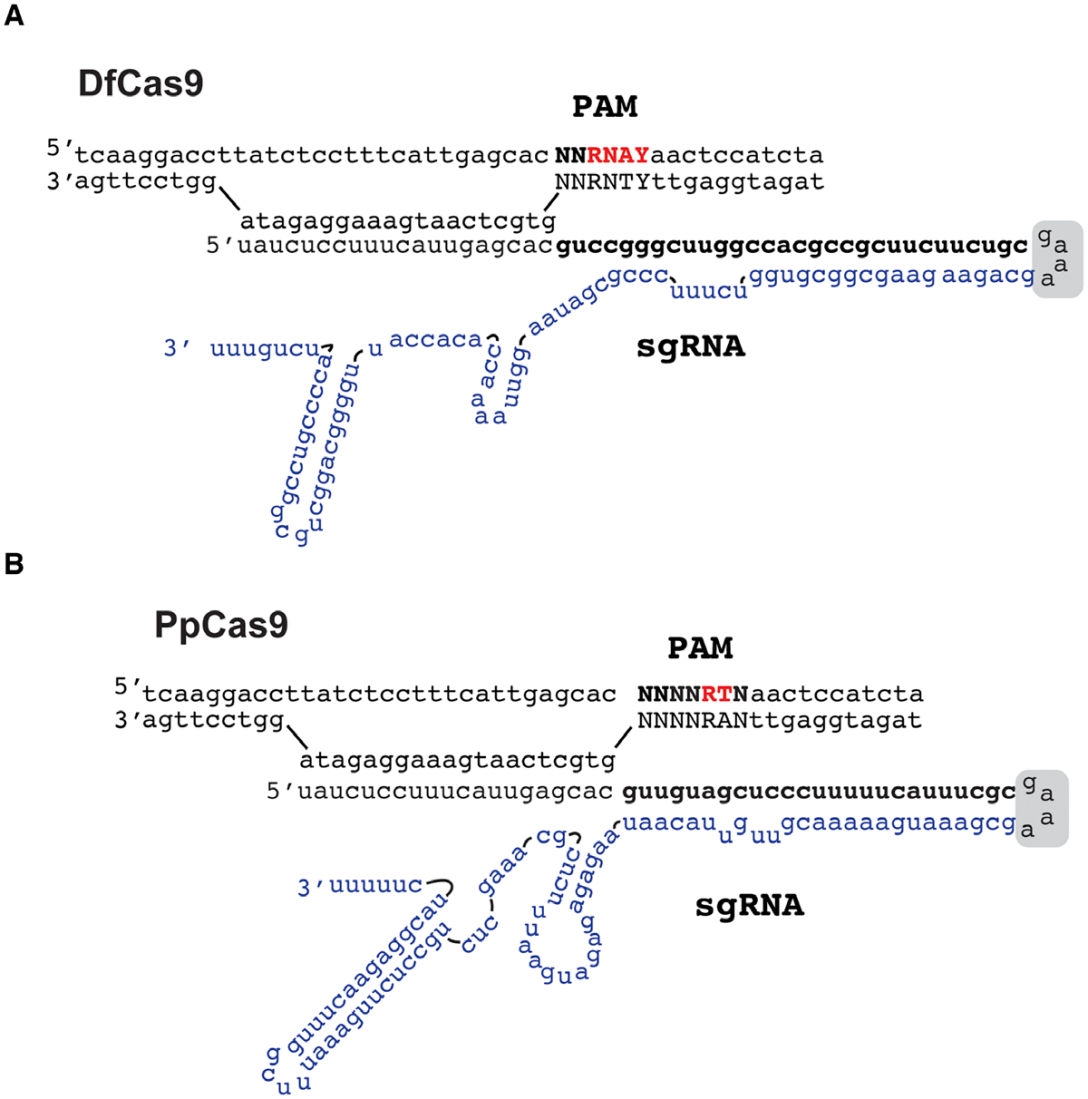
The DfCas9 and PpCas9 minimal *in vitro* DNA cleavage systems. (**A**) A scheme of recognition by the DfCas9–sgRNA complex of a DNA target flanked by 5′-NNRNAY-3′ PAM (Y stands for pyrimidines, R stands for purines). The crRNA-tracrRNA linker is indicated with a gray box. The part of sgRNA that originated from tracrRNA is shown in blue. (**B**) A scheme of recognition by the PpCas9–sgRNA complex of a DNA target flanked by 5′-NNNNRTN-3 PAM (R stands for purines). The crRNA-tracrRNA linker is indicated by a gray box. The part of sgRNA that originated from tracrRNA is shown in blue.

To facilitate the use of DfCas9 and PpCas9 as programmable nucleases we sought to design single guide RNA (sgRNAs) where crRNA and tracrRNA are fused. Several PpCas9 and DfCas9 sgRNAs variants were tested in *in vitro* DNA cleavage experiments (Supplementary File S1 and Figure S3). As a result, we determined DfCas9 and PpCas9 sgRNA forms, which supported efficient DNA cleavage *in vitro* (Figure 6).

### PpCas9 nuclease is active in human cells

**Figure 7.**
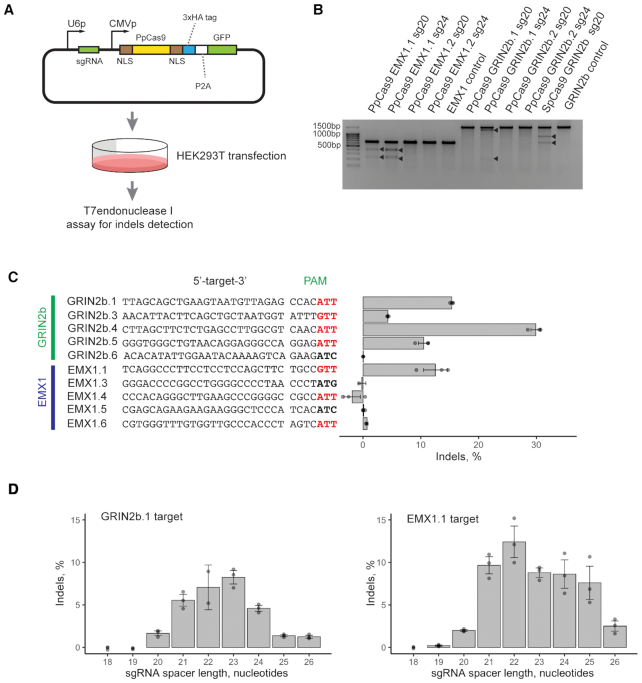
PpCas9 nuclease activity in human HEK293T cells. (**A**) Scheme of the PpCas9 nuclease activity assessment experiment. Above: A scheme showing design of a plasmid used for PpCas9 gene and sgRNAs expression. The PpCas9 gene is shown as a yellow rectangle, NLS (nuclear localization signals) as brown rectangles, GFP gene as a green rectangle. CMV promoter and U6 promoters are indicated with black arrows. The sgRNA coding sequence is shown as a green rectangle. The plasmid was transfected into HEK293T cells and genomic DNA was extracted from a heterogeneous population of modified and unmodified cells for indel frequency assessment through HTS of the targeted region or *in vitro* assay with T7 endonuclease I. (**B**) Results of T7 endonuclease I indel detection assay showing PpCas9-mediated cleavage of EMX1 and GRIN2b genes in HEK293T genome. (**C**) PpCas9 indel formation efficiency at different genomic sites. Left: genomic DNA target sites with corresponding PAM sequences. 5′-NNNNRTT-3′ PAM are shown in red. Right: indel frequency estimated by HTS analysis. Mean values and standard deviations obtained from three biological replicas are shown. (**D**) The influence of sgRNA spacer length on PpCas9-mediated indel formation efficiency in EMX1 and GRIN2b genes. HEK293T cells were transfected with PpCas9_sgRNA plasmids (as in panel A) coding for sgRNAs with different lengths of spacer segments. Left: results for the GRIN2b.1 target, right: for the EMX1.1 target. Mean values and standard deviations obtained from three biological replicas are shown.

Next, we tested DfCas9 and PpCas9 activity in human cells. Codon optimized DfCas9 and PpCas9 genes, as well as the SpCas9 gene, which was used as a positive control, were cloned into plasmid vectors under regulation of constitutive CMV promoter. A GFP coding sequence was fused to in frame with nuclease open reading frames through a sequence encoding P2A self-cleaving peptide. Appropriate sgRNA coding sequences were introduced into plasmids upstream of nuclease genes under the control of the U6 promoter (Figure 7A). PpCas9 and DfCas9 were targeted to two human genes, EMX1 and GRIN2b, with two targeting sites tried in each gene (Supplementary Table S4). SpCas9 was targeted to the GRIN2b gene only.

Extension of complementary region between a target sequence and the guide RNA spacer part increased the genome editing activity for several Type II-C nucleases (16,21). Given this, for each site we used sgRNAs with spacer sequences of two lengths: 20 and 24 nt. In the case of SpCas9 only sgRNAs with optimal 20 nt spacer sequences were used. Plasmids were transfected into human HEK293T cells and production of recombinant Cas9 proteins was confirmed by western blot analysis (Supplementary Figure S4). The efficiency of transfection was about 30%. Two days after transfection genomic DNA was extracted from a heterogeneous population of modified and unmodified cells and indel formation (nucleotides insertion or deletions) was assessed using the T7 endonuclease I detection assay.

No genome modification was detected in cells transfected with DfCas9 (data not shown). In contrast, PpCas9 introduced indels in EMX1.1 and GRIN2b.1 sites (Figure 7B) but failed to modify the EMX1.2 and GRIN2b.2 sites. Where cleavages were observed, sgRNAs with spacer sequences of 24 nt were more effective, in agreement with data for other Type II-C effectors (16,21).

Next, the activity of PpCas9 at additional sites in the GRIN2b or EMX1 genes flanked by 5′-NNNNRTN-3′ was tested using sgRNAs with 24 nt spacer-derived part (Figure 7B) (in total 12 different sites were tested). PpCas9 efficiently introduced indels in GRIN2b.1–5 and EMX1.1 targets (the significance of modification compared to negative control was indicated by one-tailed Mann–Whitney U test; U = 0, *P*-value = 0.05). Examples of indels generated can be found in Supplementary Figure S5. Interestingly, PpCas9 modified more targets in GRIN2b compared to EMX1, possibly due to DNA methylation or other factors, which can impede the binding of the nuclease to genomic DNA in the latter gene. Although the *in vitro* DNA cleavage experiments demonstrated only a slight preference for T in the seventh PAM position, in human cells PpCas9 efficiently cleaved most of the targets flanked by the 5′-NNNNRTT-3′ PAM and failed to cleave targets flanked by PAMs with no T in the seventh position. This suggests that T in the seventh PAM position, although non-essential for *in vitro* DNA cleavage, is important for DNA recognition in human cells.

The length requirements of PpCas9 sgRNA spacer needed for optimal genome editing were investigated in further detail. HEK293T cells were transfected by PpCas9 carrying plasmids analogous to those described above but coding for sgRNAs of different spacer length targeting two sites in the human genome. The assessment of DNA modification efficiency was performed using HTS sequencing of targeted sites. The results showed that PpCas9 efficiently introduces double-stranded breaks in genomic DNA with sgRNAs of 21–24 nt spacer length (Figure 7D). The highest levels of genome modification were achieved when sgRNAs with 22–23 nt spacers were used.

### The specificity of PpCas9

**Figure 8.**
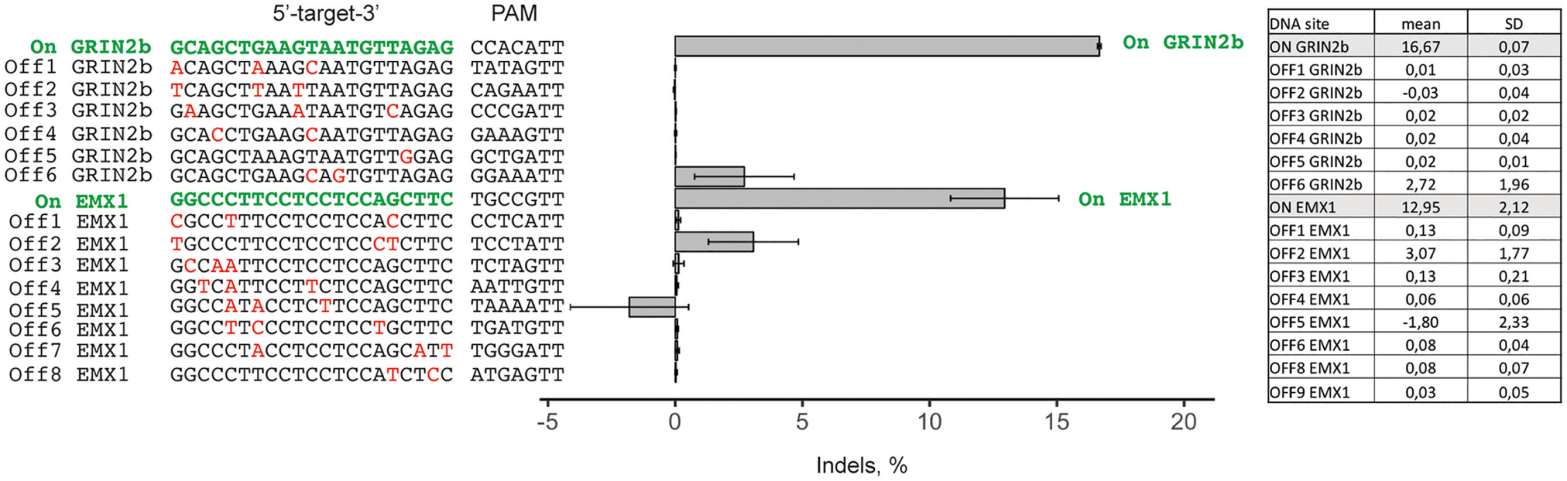
The specificity of genomic DNA cleavage by PpCas9. The indel frequency at two on-target as well as at corresponding off-target sequences was assessed by targeted amplicon sequencing of genomic DNA of HEK293T cells transfected with plasmids carrying the PpCas9 genome editing system. Left: Sequences of on-target sites (in green) and off-target sites are shown. Mismatches in off-target sequences are shown in red. Right: Frequencies of indel formation in each site (mean values and standard deviations obtained from three replicas are shown in a table).

The use of Cas nucleases in biotechnology requires that they recognize their target specifically. We conducted a preliminary study of PpCas9 cleavage specificity. First, we assessed *in vitro* how mismatches between the spacer fragment of an sgRNA and the cognate protospacer sequence affect cleavage. Similarly to other Cas9 nucleases (13,15), mismatches in PAM-proximal region of the protospacer decreased the PpCas9 target cleavage activity (Supplementary Figure S6 and Table S6). Next, we assessed PpCas9 off-target activity in eukaryotic cells. As on-targets we chose two DNA sites in EMX1 and GRIN2b genes, which were efficiently cleaved by PpCas9 in previous experiments (EMX1.1 and GRIN2b.1). HEK293T cells were transfected with plasmids carrying the PpCas9 genome-editing system targeting these sites. In this experiment we directed PpCas9 to on-target sequences using sgRNAs with spacer lengths of 21 nt. Such sgRNAs provide sufficient DNA cleavage efficiency and allow to identify more potential off-target sequences than sgRNAs with longer, 22–23 nt spacer segments. Three days post-transfection genomic DNA was extracted and indel frequency at the on-target as well as at likely off-target sites (sequences differing by up to 3 nt from on-target sites) was assessed by targeted amplicon high-throughput sequencing. The computational analysis using CRISPResso2 and further statistical analysis using one-tailed Mann–Whitney U test indicated significant modifications compared to negative controls only at on-target GRIN2b and EMX1 sites, and on an EMX1 off-target site 2 (U = 0, *P*-value = 0.05) (Figure 8). In case of the EMX1 off-target site 2 all identified differences were single nucleotide substitutions, which could have resulted from sequencing errors rather than true off-target activity. In contrast, in on-target sites PpCas9 generated indels of different lengths. The modification of remaining off-target sites compared to negative control was statistically insignificant (one-tailed Mann–Whitney U test; U > 0, *P*-value > 0.05). Based on these preliminary results, we conclude that PpCas9 can be considered as a promising nuclease in terms of its specificity though additional studies using Digenome-seq, BLISS or other methods should be used to fully estimate its off-targeting activity.

### PpCas9 is closely related to NmeCas9

**Figure 9.**
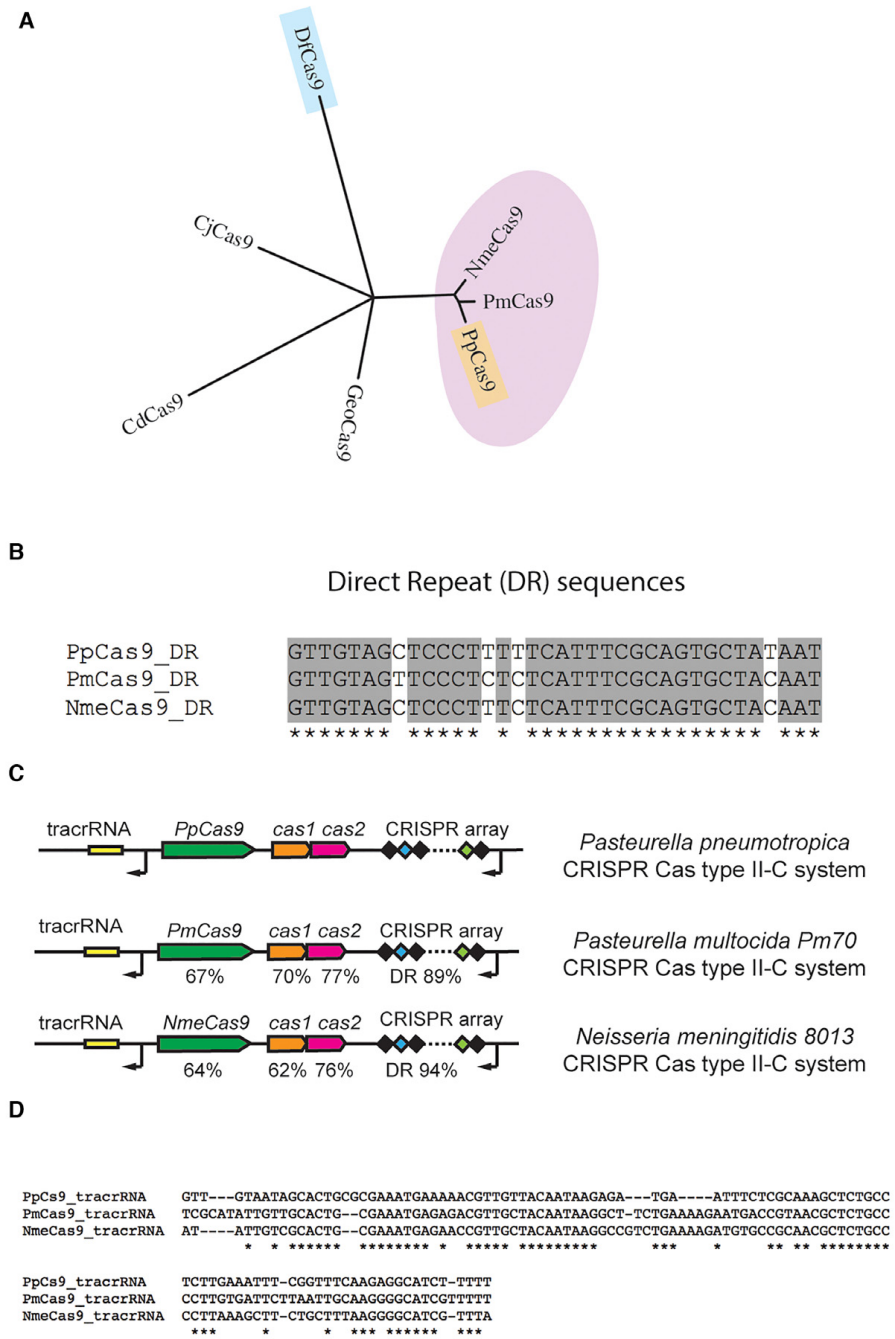
PpCas9 is closely related to NmeCas9 and PmCas9. (**A**) An unrooted phylogenetic tree showing the relationship of PpCas9 to NmeCas9 and PmCas9. Nodes with strong statistical support (bootstrap values > 80%) are shown. (**B**) An alignment of PpCas9, PmCas9 and NmeCas9 direct repeat sequences. Identical nucleotides are indicated with asterisks. (**C**) Comparison of CRISPR-Cas Type II-C loci from *Pasteurella pneumotropica*, *Pasteurella multocida Pm70* and *Neisseria meningitidis*. The percent of amino acid identities with *P. pneumotropica* sequences for Cas1, Cas2 and Cas9 orthologues and nucleotide identities for DRs are shown. (**D**) PpCas9, PmCas9 and NmeCas9 tracrRNA coding sequences alignment. Identical nucleotides are indicated with asterisks.

While both DfCas9 and PpCas9 are active *in vitro*, only PpCas9 demonstrated activity in eukaryotic cells. Hence, we decided to compare DfCas9 and PpCas9 amino acid sequences with other Type II-C orthologues active in human cells. A phylogenetic tree of PpCas9 and DfCas9 with CjCas9, CdCas9, NmeCas9 and GeoCas9 showed that while DfCas9 is not close to these active nucleases, PpCas9 is fairly close to the Cas9 enzyme from *N. meningitidis*, NmeCas9 (Figure 9A, Supplementary Figure S7 and File S4). NmeCas9 along with its highly similar orthologue Nme2Cas9 (a Type II-C effector from another strain of *N. meningitidis)* are highly efficient in human cells and considered as promising candidates for biomedical applications. Despite the sequence similarity, PpCas9, NmeCas9 and Nme2Cas9 have different PAM requirements (5′-NNNNRTT-3′ for PpCas9; 5′-NNNNGMTT-3′ for NmeCas9 and 5′-NNNNCC-3′ for Nme2Cas9).

A Blast search also revealed that PpCas9 is highly similar to PmCas9, a nuclease from *Pasteurella multocida Pm70* whose PAM was bioinformatically determined by Fonfara *et al.* (35) to be 5′-GNNNCNNA-3′. Although preliminary DNA cleavage experiments showed that PmCas9 is active *in vitro*, no information on activity of this nuclease in human cells is available to date.

A comparison of Type II-C *P. pneumotropica*, *P. multocida Pm70* and *N. meningitidis* DR sequences, tracrRNAs and *cas* genes responsible for adaptation showed that they are also highly similar (Figure 9B–D and Supplementary File S5).

## DISCUSSION

CRISPR-Cas nucleases of small size are promising for biomedicine as their genes can be delivered in eukaryotic organisms using size-restricted vectors, such as AAV (16,33,36). Only several Cas9 orthologues smaller than SpCas9 were characterized to date and shown to be active in human cells: SaCas9, CjeCas9, GeoCas9, CdCas9, SauriCas9, NmeCas9 and Nme2Cas9 (16–18,20–22,31,33). Most of them have long complicated PAM sequences (5′-NNGRRT-3′, 5′-NNNNRYAC-3′, 5′-NNNNCRAA-3′ and 5′-NNRHHHY-3′ for SaCas9, CjeCas9, GeoCas9, CdCas9, correspondingly). The only known exceptions are Nme2Cas9 and SauriCas9 which require the 5′-NNNNCC-3′and 5′-NNGG-3′ PAMs, correspondingly. These nucleases were found relatively recently and demonstrated high efficiency of editing in human cells (17,18). In this work, we characterized two small sized Type II-C CRISPR-Cas effectors from *Defluviimonas sp.20V17* and *P. pneumotropica*, DfCas9 and PpCas9. We show that both these proteins are active nucleases, which are able to cleave DNA targets *in vitro*. In addition, we show that the PpCas9 nuclease, which requires a novel PAM sequence 5′-NNNNRTT-3′ (5′-NNNNRT-3′ for *in vitro* DNA cleavage) possess a DNA cleavage activity in human cells (Supplementary Figure S8).

At 1055 amino acids, PpCas9 is similar to small Cas9 orthologues SaCas9, CjCas9, Nme2Cas9 and SauriCas9 (1053, 984, 1082 and 1061 amino acids, correspondingly), and thus its gene potentially can be delivered along with sgRNA coding sequences via a single size-restricted vector such as AAV. We therefore submit that PpCas9 could be used as a genome-editing instrument, although additional studies of its efficiency should be conducted. Indeed, PpCas9 cleaved DNA targets in the GRIN2b gene more efficiently than in EMX1, which may reflect its preference for certain genome methylation patterns, possible variations of PAM preferences *in vitro* and in eukaryotes, folding of DNA and/or other factors that remain to be determined. This selectivity to DNA targets observed in human cells reduces the range of possible PpCas9 genomic targets. The nature of this bias is a subject of further studies.

While the PpCas9 specificity also should be studied in more detail, the preliminary data obtained in this study demonstrate that this nuclease is unlikely to possess high off-targeting activity.

Interestingly, PpCas9 is quite similar in its amino acid sequence to NmeCas9 and Nme2Cas9 proteins, which both are highly efficient in human cells. The sequence similarity is observed in all components of CRISPR-PpCas9 and CRIPSR-NmeCas9 systems: the DR sequences, the tracrRNAs, and the *cas* genes, although the host bacteria belong to different classes (*Gammaproteobacteria* and *Betaproteobacteria*, respectively). The CRISPR-PpCas9 locus components are also very similar to a Type II-C system from *P. multocida Pm70*. One can speculate that the PmCas9 effector protein, which demonstrated activity *in vitro* (35), could also be active in human cells.

The *Defluviimonas sp. 20V17* nuclease DfCas9, also characterized in this study, did not show observable genome-editing activity in human cells. Yet, this small size nuclease with distinct PAM requirements is active at least in *E. coli* and thus may also find biotechnological applications in microbial engineering.

## DATA AVAILABILITY

Raw sequencing data have been deposited with the National Center for Biotechnology Information Sequence Read Archive under BioProject ID PRJNA629762 and PRJNA629763.

## Supplementary Material

gkaa998_Supplemental_Files
