# Supplementary material for: PpCas9 from Pasteurella pneumotropica — a compact Type II-C Cas9 ortholog active in human cells
Source: Nucleic Acids Res. 2020 Nov 5;48(21):12297–309. doi: 10.1093/nar/gkaa998 (PMC7708072; doi:10.1093/nar/gkaa998)
Supplement: gkaa998_Supplemental_Files [file gkaa998_supplemental_files.zip › Supplementary File S1_R2.pdf]

Supplementary File S1

Supplementary Table S1. DNA sequences used in this study.

locus_DfCas9_F primer	atctcaagaagatcatcttattaatcagataaaatatttctagaTGGA CGGAACACAGGGGCCGGACGTG
locus_DfCas9_R primer	caatttaactgtgataaactaccgcattaaagcttATCACTGAA CATGTCCCTGATGCTGATCGAG
pACYC184_DfCas9_locus (plasmid used for experiments in bacteria; native, non-codon optimized PpCas9 gene sequence)	https://benchling.com/s/seq-2X6d3vv4B2dM4qajqFuk
CRISPR-PpCas9 locus (native, non-codon optimized PpCas9 gene sequence)	https://benchling.com/s/seq-2rHKjZaY71sBPevLcrXM
DfCas9_R primer	gagtgcggccgcaagcttAACTGTCCCATGCGGGATC GTGTGAACCCGCCCCAATTCGTCGATCCGCAC
DfCas9_F primer	gaaggagatatacatatgATGTATCGTTTCGCTTTCGA CCTCGGAACCAAC
pET21a_DfCas9 plasmid	https://benchling.com/s/seq-Rcl0JPmGbUo5J2ef8tQq
pET21a_PpCas9 plasmid	https://benchling.com/s/seq-RSWQPFNAnE05Xg3X8gkV
7N_PUC19_DfCas9_library	https://benchling.com/s/seq-bt807FquL2f5Zdbb9kgy
PUC19_R primer	agcttggcgtaatcatggtcatag
PUC19_F primer	cccgggtaccgagctcga
Library_F primer	ctatgaccatgattacgccaagctgatcatgatcgacatgatcc cgaaggtctaNNNNNNNcccgggtaccgagctcga
Library_R primer	tcgagctcggtacccggg
M13_f primer	GTTGTAAAACGACGGCCAGTG
M13_r primer	AGCGGATAACAATTTCACACAGGA
1592 bp DNA fragment used for *in vitro* reactions	https://benchling.com/s/seq-atpZZkfQvY1aiGMgr25K
U6_sgRNA_BsmBI_CMV_PpCas9_P2A_GFP	https://benchling.com/s/seq-zFrQDDaVHSsxwMuyNuf8
U6_sgRNA_BsmBI_CMV_SpCas9_P2A_GFP	https://benchling.com/s/seq-g4bqSa99fdbF7JPn7eJp
GRIN2b_T7_endoI_fragment	https://benchling.com/s/seq-FCZIYuNF0rPKw4wXKBvD
EMX1_T7_endoI_fragment	https://benchling.com/s/seq-BuPF1uTcGA3WMTyOGtf0

Supplementary Table S2. RNA sequences used in *in vitro* experiments.

PpCas9 crRNA	**GGG**tatctcctttcattgagcacGTTGTAGCTCCCTTTTTCATTTCGC
PpCas9 tracrRNA	**GG**GCGAAATGAAAAACGTTGTTACAATAAGAGATGAATTTCTCGCAAAGCT CTGCCTCTTGAAATTTCGGTTTCAAGAGGCATCTTTTT
DfCas9 crRNA	**GGG**tatctcctttcattgagcacGTCCGGGCTTGGCCACGCCGCTTCTTCTGCTAGGAT
DfCas9 tracrRNA	**GGG**TCCTAGCAGAAGAAGCGGCGTGGTCTTTCCCGCGATAAGGTTAAAACCACACCAT TGGGGCAGGCTGCGGCCTGCCCCATCTGTTT
DfCas9 sgRNA 1 (Fig. S2)	**GGG**TATCTCCTTTCATTGAGCACGTCCGGGCTTGGCCACGCCGCTTCGAAA GAAGCGGCGTGGTCTTTCCCGCGATAAGGTTAAAACCACACCATTGGGGCAG GCTGCGGCCTGCCCCATCTGTTT
DfCas9 sgRNA 2	**GGG**TATCTCCTTTCATTGAGCACGTCCGGGCTTGGCCACGCGAAAGCGTGGTC TTTCCCGCGATAAGGTTAAAACCACACCATTGGGGCAGGCTGCGGCCTGCCCCA

(Fig. S2)	TCTGTTT
DfCas9 sgRNA 3 (Fig. S2)	**GGG**TATCTCCTTTCATTGAGCACGTCCGGGCTTGGCCACGCCGCTTCTTCTGCG AAAGCAGAAGAAGCGGCGTGGTCTTTCCCGCGATAAGGTTAAAACCACACCATTG GGGCAGGCTGCGGCCTGCCCCATCTGTTT
PpCas9 sgRNA1 (Fig. S2)	**GGG**TATCTCCTTTCATTGAGCACGTTGTAGCTCCCTTTTTCATTTCGCGAAAGCGAAATG AAAAACGTTGTTACAATAAGAGATGAATTTCTCGCAAAGCTCTGCCTCTTGAAATTTCGG TTTCAAGAGGCATCTTTTT
PpCas9 sgRNA2 (Fig. S2)	**GGG**TATCTCCTTTCATTGAGCACGTTGTAGCTCCCTTTTTCATTTCGCAGTGCTATAATGAA AATTATAGCACTGCGAAATGAAAAACGTTGTTACAATAAGAGATGAATTTCTCGCAAAGC TCTGCCTCTTGAAATTTCGGTTTCAAGAGGCATCTTTTT

Spacer-derived sequences are shown on cyan background (note that for DNA cleavage in HEK293T, sgRNA with spacers segments of 22-23 nt are more effective). The 5'-terminal G-nucleotides arising during synthesis by T7 RNA polymerase are shown in bold.

Supplementary Table S3. DNA targets in a 1592 bp fragment used in *in vitro* DNA cleavage experiments.

PpCas9 grin2b targets	protospacer	PAM
target 1	ATATTGGAATACAAAAGTCA	GAAGATCt
target 2	GTGGGCACTTCCGACGAGGT	GGCCATCa
target 3	GCCTTGGCGTCAACATTTAT	AAAAATGg
target 4	GGCAATGTTTTAAAACTACT	AGTCATGt
target 5	CAAGAATGCAGGGCTTGTGT	ACTTATAg
target 6	CTCTGCCTGTAGCTGCCAAT	GACTATAg
target 7	TGAGCAGAAAAACGTGCTCA	CTTTGTCt
target 8	TGGTGCTCAATGAAAGGAGA	TAAGGTCc
target 9	CTAGCCTCTTCTAAGACAGG	TTACGTGa
target 10	ATCCTCGTGGGCACTTCCGA	CGAGGTGg
target 11	TATTTTATTCCCTACTGGGT	CATTGTAa
target 12	AGGAGATAAGGTCCTTGAAT	TGCAGTAt
DfCas9 grin2b targets	protospacer	PAM
target 1	TTGTAGTTTAATATTAAGAG	CCAGATAc
target 2	CTCAATGAGGACAACAGCCA	GAAAATTc
target 3	CAGAACATGGGCTGGATAAA	CTGGATGt
target 4	ATCAATTAAGTGAAACAAAC	CTGAACCa
target 5	CTCTGCAGAATGAGAGAAAA	TGAAACTt
target 6	ATCTTCTGACTTTTGTATTC	CAATATGt
target 7	GCTGCCAATGACTATAGCAA	TAGCACCt
target 8	CTTCAGCCCAAGAACAGTAC	AAGGGTGg
target 9	TATTAAACTACAAGAACAAC	TGATACAt

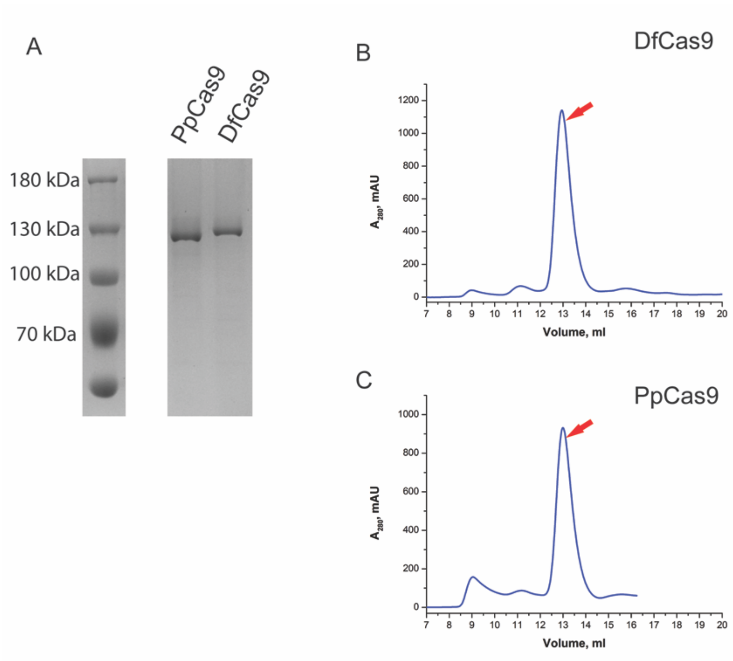

Supplementary Figure S1. Purification of DfCas9 and PpCas9.

A. An SDS gel showing purified recombinant PpCas9 and DfCas9.
B. Size exclusion chromatography elution profile of DfCas9. A monomer fraction is marked with a red arrow.
C. Size exclusion chromatography elution profile of PpCas9. A monomer fraction is marked with a red arrow.

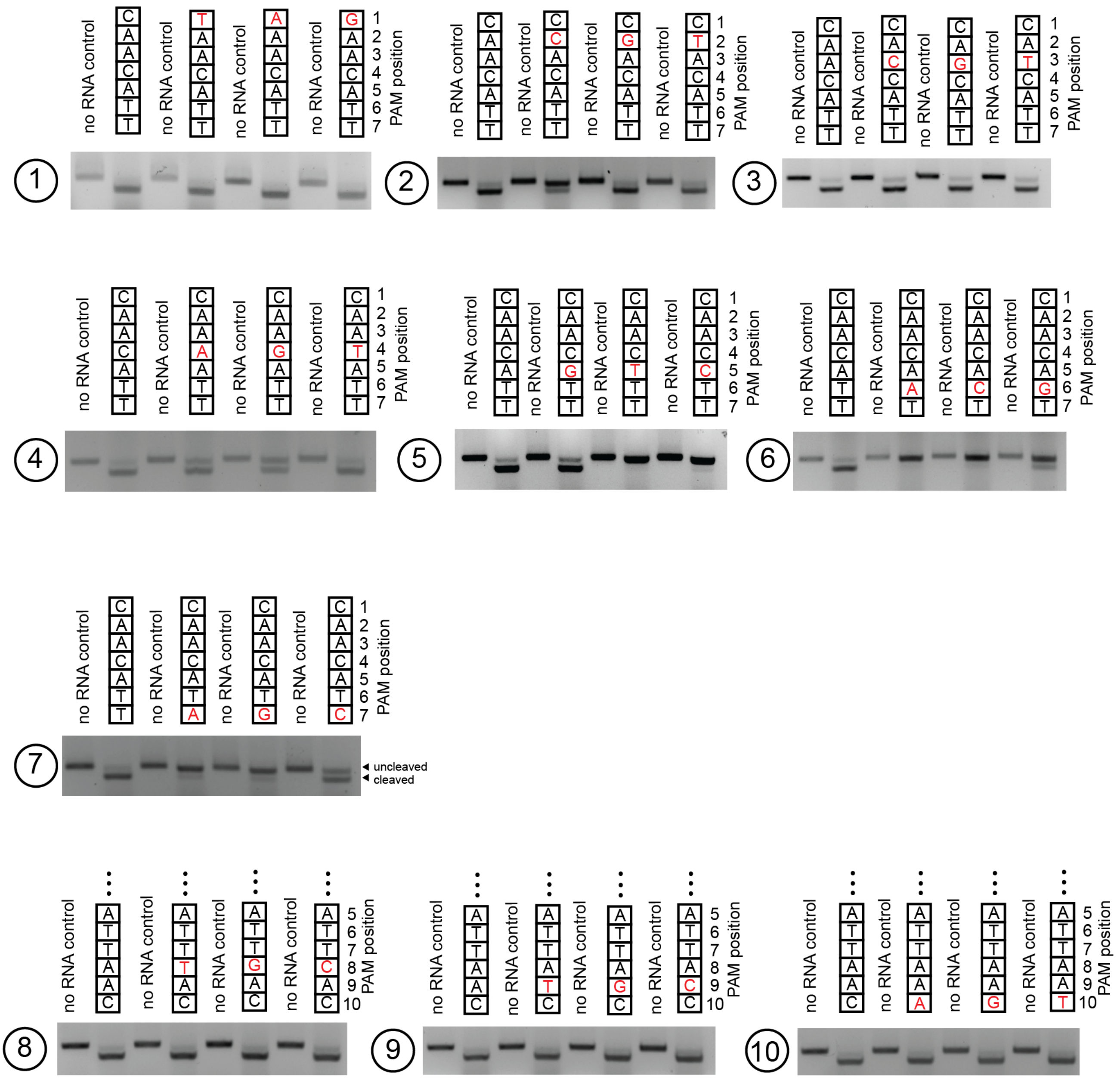

Supplementary Figure S2. The effect of single-nucleotide substitutions in PAM on PpCas9 DNA cleavage efficiency.

The agarose gels showing the results of electrophoretic separation of targets with PAM sequences shown at the top after incubation for 30 minutes with the PpCas9 effector complex are presented. Bands corresponding to cleaved and uncleaved DNA fragments are indicated.

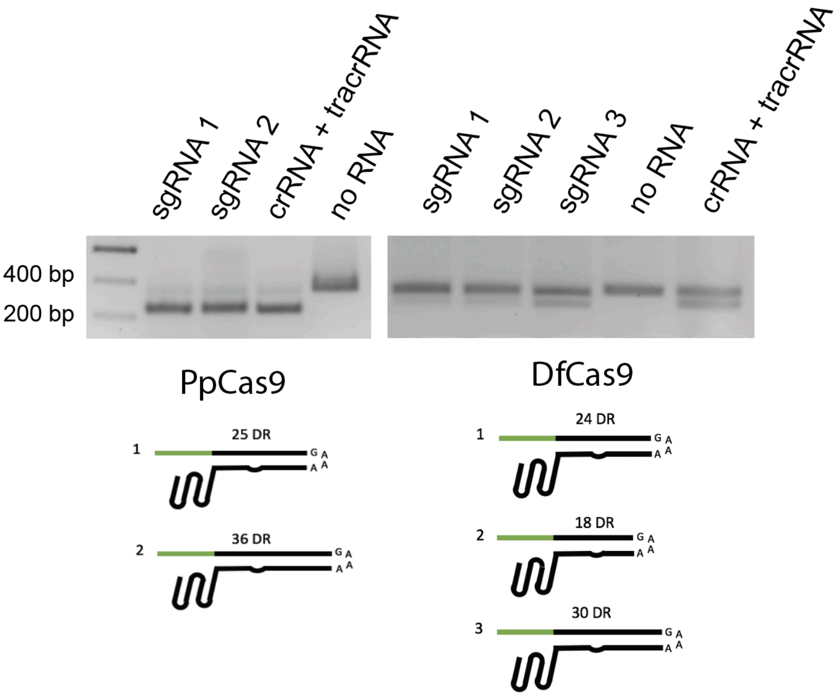

Supplementary Figure S3. PpCas9 and DfCas9 sgRNA design.

Several variants of sgRNA were used for PpCas9 (left panel) or DfCas9 (right panel) DNA cleavage reactions *in vitro*. To estimate DNA cleavage efficiency reactions products were loaded on 1.5% agarose gel and results of reaction products separation are shown at the top.

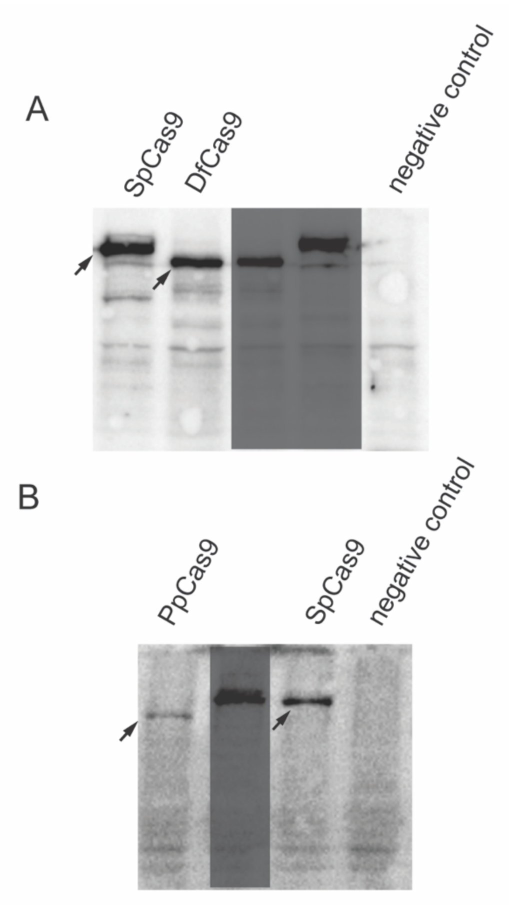

Supplementary Figure S4. PpCas9 and DfCas9 expression in eukaryotic cells.

HEK293T cells were transfected with plasmids U6_sgRNA_BsmBI_CMV_DfCas9_P2A_GFP (A) or U6_sgRNA_BsmBI_CMV_PpCas9_P2A_GFP (B) and U6_sgRNA_BsmBI_CMV_SpCas9_P2A_GFP. Two days after transfection cell lysate was prepared and Western blotting was performed using antibodies against 3xHA-tag incorporated at the C-terminal ends of effectors. Bands corresponding to Cas proteins are indicated by black arrows.

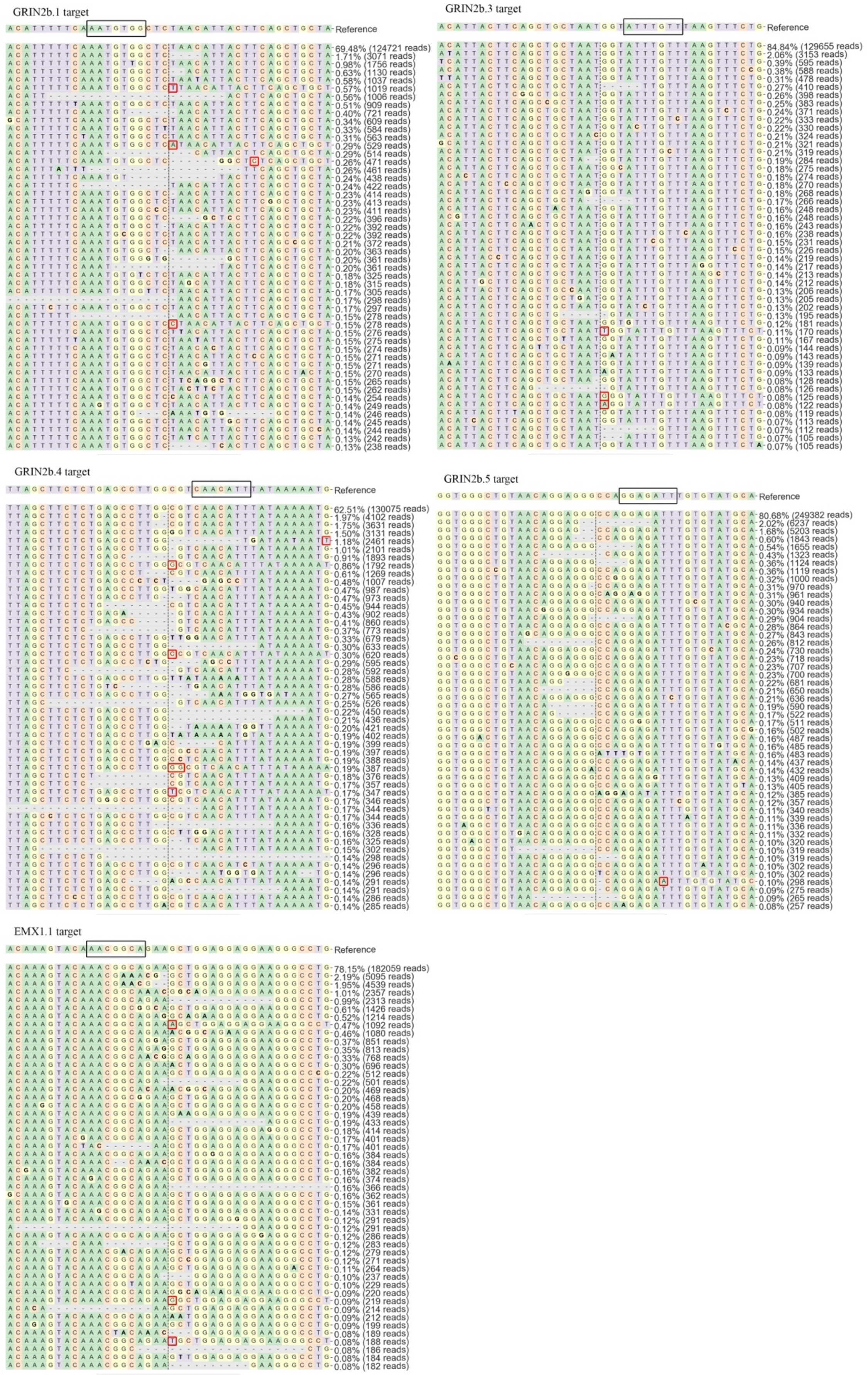

Supplementary Figure S5. Indels generated by PpCas9 in genome targets.

The most frequent alleles identified by CRISPResso2 in GRIN2b.1, GRIN2b.3, GRIN2b.4, GRIN2b.5, and EMX1.1 targets in cells transfected by plasmids carrying CRISPR-PpCas9 system

targeting the corresponding sites. Substitutions are shown in bold font; red rectangles indicate inserted sequences and horizontal dashed lines indicate deletions. The putative DNA cleavage position is shown by vertical dashed line. Seven-nucleotide PAMs are shown by black rectangles.

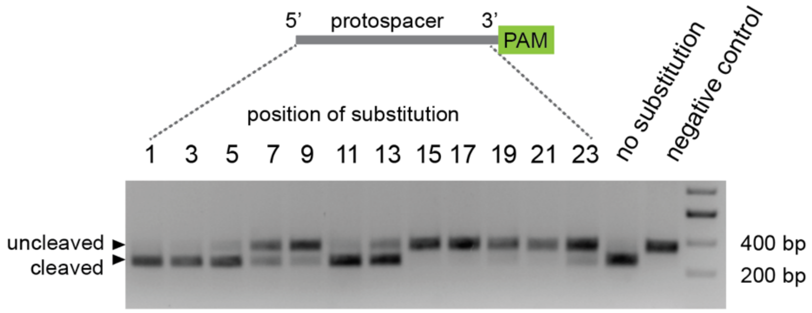

Supplementary Figure S6. Effect of mismatches in protospacer sequence on PpCas9 DNA cleavage activity *in vitro*.

PpCas9 was targeted *in vitro* to linear DNA fragments with purine to pyrimidine or pyrimidine to purine single-nucleotide substitutions in the protospacer. An agarose gel showing the results of electrophoretic separation of cleavage products of targets with substitutions in indicated positions of protospacer (counting from the 5'-end) is presented. Bands corresponding to cleaved and uncleaved DNA fragments are indicated.

Supplementary Figure S7

```
NmeCas9    1  MAAFKPNSINYILGLDIGIASVGWAMVEIDEEENPIRLIDLGVRVFERAEVPKTGDSLAM
PpCas9     1  MQ---NNPLNYILGLDLGIASIGWAVVEIDEESSPIRLIDVGVRTFERAEVAKTGESLAL
              RuvC I | Rec lobe

NmeCas9   61  ARRLARSVRRLTRRRAHRLLRTRRLLKREGVLQAANFDENGLIKSLPNTPWQLRAAALDR
PpCas9    58  SRRLARSSRRLIKRRAERLKKAKRLLKAEKILHSID-------EKLPINVWQLRVKGLKE
              Rec lobe

NmeCas9  121  KLTPLEWSAVLLHLIKHRGYLSQRKNEGETADKELGALLKGVAGNAHALQTGDFRTPAEL
PpCas9   111  KLERQEWAAVLLHLSKHRGYLSQRKNEGKSDNKELGALLSGIASNHQMLQSSEYRTPAEI
              Rec lobe

NmeCas9  181  ALNKFEKESGHIRNQRSDYSHTFSRKDLQAELILLF[illegible]KQKEFGNPHVSGGLKEGIETLLM
PpCas9   171  AVKKFQVEEGHIRNQRGSYTHTFSRLDLLAEMELLFQRQAELGNSYTSTTLLENLTALLM
              Rec lobe

NmeCas9  241  TQRPALSGDAVQKMLGHCTFEPAEPKAAKNTYTAERFIWLTKLNNLRILEQGSERPLTDT
PpCas9   231  WQKPALAGDAILKMLGKCTFEPSEYKAAKNSYSAERFVWLTKLNNLRILENGTERALNDN
              Rec lobe

NmeCas9  301  ERATLMDEPYRKSKLTYAQARKLLGLEDTAFFKGLRY-GKDN---AEASTLMEMKAYHAI
PpCas9   291  ERFALLEQPYEKSKLTYAQVRAMLALSDNAIFKGVRYLGEDKKTVESKTTLIEMKFYHQI
              Rec lobe

NmeCas9  357  SRALEKEGLKDKKSPLNLSPELQDEIGTAFSLFKTDEDITGRLKDRIQPEILEALLKHIS
PpCas9   351  RKTLGSAELKKEWNELKGNSDLLDEIGTAFSLYKTDDDICRYLEGKLPERVLNALLENLN
              Rec lobe

NmeCas9  417  FDKFVQISLKALRRIVPLMEQGKRYDEACAEIYGDHYGKKNTEEKIYLPPIPADEIRNPV
PpCas9   411  FDKFIQLSLKALHQILPLMLQGQRYDEAVSAIYGDHYGKKSTETTRLLPTIPADEIRNPV
              Rec lobe

NmeCas9  477  VLRALSQARKVINGVVRRYGSPARIHIETAREVGKSFKDRKEIEKRQEENRKDREKAAAK
PpCas9   471  VLRTLTQARKVINAVVRLYGSPARIHIETAREVGKSYQDRKKLEKQQEDNRKQRESAVKK
              RuvC II | HNH

NmeCas9  537  FREYFPNFVGEPKSKDILKLRLYEQQHGKCLYSGKEINLGRLNEKGYVEIDHALPFSRTW
PpCas9   531  FKEMFPHFVGEPKGKDILKMRLYELQQAKCLYSGKSLELHRLLEKGYVEVDHALPFSRTW
              HNH

NmeCas9  597  DDSFNNKVLVLGSENQNKGNQTPYEYFNGKDNSREWQEFKARVETSRFPRSKKQRILLQK
PpCas9   591  DDSFNNKVLVLANENQNKGNLTPYEWLDGKNNSERWQHFVVRVQTSGFSYAKKQRILNHK
              HNH

NmeCas9  657  FDEDGFKERNLNDTRYVNRFLCQFVADRMRLTGKGKKRVFASNGQITNLLRGFWGLRKVR
PpCas9   651  LDEKGFIERNLNDTRYVARFLCNFIADNMLLVGKGKRNVFASNGQITALLRHRWGLQKVR
              HNH | RuvC III

NmeCas9  717  AENDRHHALDAVVVACSTVAMQQKITRFVRYKEMNAFDGKTIDKETGEVLHQKTHFPQPW
PpCas9   711  EQNDRHHALDAVVVACSTVAMQQKITRFVRYNEGNVFSGERIDRETGEIIP--LHFPSPW
              RuvC III

NmeCas9  777  EFFAQEVMIRVFGKPDGKPEFEEADTLEKLRTLLAEKLSSRPEAVHEYVTPLFVSRAPNR
PpCas9   769  AFFKENVEIRIFSEN--------------PKLELENRLPDYPQYNHEWVQPLFVSRMPTR
              RuvC III

NmeCas9  837  KMSGQGHMETVKSAKRLDEGVSVLRVPLTQLKLKDLEKMVNREREPKLYEALKARLEAHK
PpCas9   815  KMTGQGHMETVKSAKRLNEGLSVLKVPLTQLKLSDLERMVNRDREIALYESLKARLEQFG
              WED

NmeCas9  897  DDPAKAFAEPFYKYDKAGNRTQQVKAVRVEQVQKTGVWVRNHNGIADNATMVRVDVFEKG
PpCas9   875  NDPAKAFAEPFYKKGG-----ALVKAVRLEQTQKSGVLVRDGNGVADNASMVRVDVFTKG
              WED | PI
```

```
NmeCas9   957 DKYYLVPIYSWQVAKGILPDRAVVQGKDEEDWQLIDDSFNFKFSLHPNDLVEVITKKARM
PpCas9    930 GKYFLVPIYTWQVAKGILPNRAATQGKDENDWDIMDEMATFQFSLCQNDLIKLVTKKKTI
```

PI

```
NmeCas9  1017 FGYFASCHRGTGNINIRIHDLDHKIGKNGILEGIGVKTALSFQKYQIDELGKEIRPCRLK
PpCas9    990 FGYFNGLNRATSNINIKEHDLDKSKGKLGIYLEVGVKLAISLEKYQ[illegible]DELGKNIRPCRPT
```

PI

```
NmeCas9  1077 KRPPVR
PpCas9   1050 KRQHVR
```

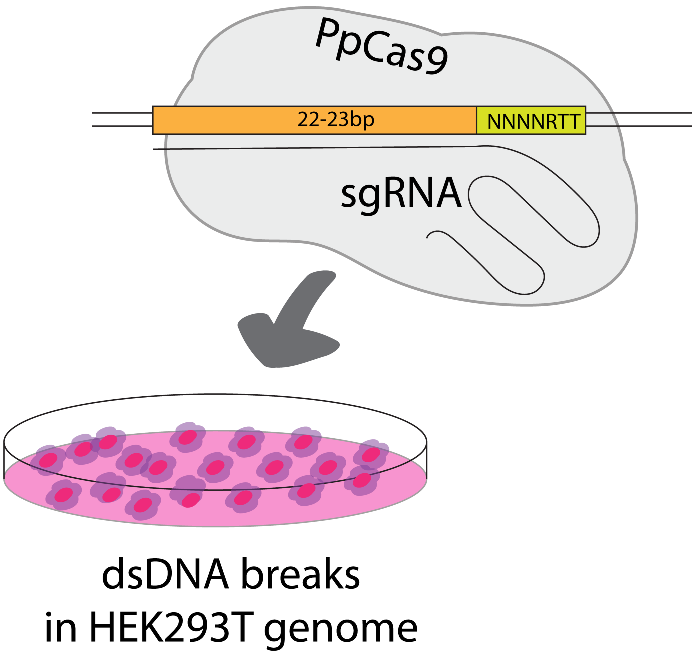

Supplementary Figure S8. PpCas9 requires sgRNAs with 22-23 nt spacer segments for recognition of DNA targets with 5’-NNNNRTT-3’ PAM sequence in the HEK293T genome.

Supplementary Table S4. DNA targets in human cells genome used in this study.

PpCas9 grin2b targets	Protospacer (target site)	PAM
PpCas9 GRIN2b 1 sg20	CAGCTGAAGTAATGTTAGAG	CCACATT
PpCas9 GRIN2b 1 sg24	TTAGCAGCTGAAGTAATGTTAGAG	CCACATT
PpCas9 GRIN2b 2 sg20	AATAAGAAAAACATTATTAT	CACCATT
PpCas9 GRIN2b 2 sg24	ATAAAATAAGAAAAACATTATTAT	CACCATT
PpCas9 EMX1 1 sg20	GCCCTTCCTCCTCCAGCTTC	TGCCGTT
PpCas9 EMX1 1 sg24	TCAGGCCCTTCCTCCTCCAGCTTC	TGCCGTT
PpCas9 EMX1 2 sg20	GGAGGTGACATCGATGTCCT	CCCCATT
PpCas9 EMX1 2 sg24	CATTGGAGGTGACATCGATGTCCT	CCCCATT
SpCas9 GRIN2b sg20	ACCTTTTATTGCCTTGTTCA	AGG

Supplementary Table S5. First-Round PCR primers used for In-Del frequency analysis.

GRIN2b_1_target_different_ spacer_lengths_F	CTCTTTCCCTACACGACGCTCTTCCGATCTNNNN**TAC GGTATCAGTCATTTTAGGGAAGTCACG**
GRIN2b_1_target_different_ spacer_lengths_R	TCAGACGTGTGCTCTTCCGATCT**ATGTGTTCTATTA CACTACGTGGAACTGCC**
EMX_1_target_different_spacer _lengths_F	CTCTTTCCCTACACGACGCTCTTCCGATCTNNNN**CCT CCTGAGTTTCTCATCTGTGCCCCTCC**
EMX_1_target_different_spacer _lenghts_R	TCAGACGTGTGCTCTTCCGATCT**GGAGGTGACATC GATGTCCTCCCCATTGG**
GRIN2b_1_target_different_ targets_F	CTCTTTCCCTACACGACGCTCTTCCGATCTNNNN**GG GAAGTCACGACTATAGGATGGCATCAGG**
GRIN2b_1_target_different_ targets_R	TCAGACGTGTGCTCTTCCGATCT**GAACACATATTAC TCCAATCTATTTATACACC**
GRIN2b_3_target_F	CTCTTTCCCTACACGACGCTCTTCCGATCTNNNN**GG GAAGTCACGACTATAGGATGGCATCAGG**
GRIN2b_3_target_R	TCAGACGTGTGCTCTTCCGATCT**GAACACATATTAC TCCAATCTATTTATACACC**
GRIN2b_6_target_F	CTCTTTCCCTACACGACGCTCTTCCGATCTNNNN**GG TTTGTTTCACTTAATTGATTGGTTCATGG**
GRIN2b_6_target_R	TCAGACGTGTGCTCTTCCGATCT**ATTGATCCTTACA ATGACCCAGTAGGG**
GRIN2b_7_target_F	CTCTTTCCCTACACGACGCTCTTCCGATCTNNNN**CAC TTTGTCTGGCCTTGCTTTCCTTCAGC**
GRIN2b_7_target_R	TCAGACGTGTGCTCTTCCGATCT**TTGTGAGTGGTCC AGGTAGCCATGCG**
GRIN2b_8_target_F	CTCTTTCCCTACACGACGCTCTTCCGATCTNNNN**ATA ATTGGTTATATGAGAGGCAGTTCCACG**

GRIN2b_8_target_R	TCAGACGTGTGCTCTTCCGATCT**GCTAAGTGTTCTAAGACCATGAACCAAT**
EMX_1_target_different_targets_F	CTCTTTCCCTACACGACGCTCTTCCGATCTNNNN**CCCAGGTGAAGGTGTGGTTCCAGAACC**
EMX_1_target_different_targets_R	TCAGACGTGTGCTCTTCCGATCT**CAATGCGCCACCGGTTGATGTGATGG**
EMX_3_target_F	CTCTTTCCCTACACGACGCTCTTCCGATCTNNNN**CTGTGAATGTTAGACCCATGGGAGCAGC**
EMX_3_target_R	TCAGACGTGTGCTCTTCCGATCT**TCAGGCTGAGCTGAGAGCCTGATGGG**
EMX_4_target_F	CTCTTTCCCTACACGACGCTCTTCCGATCTNNNN**AGCTGGACTCTGGCCACTCCCTGG**
EMX_4_target_R	TCAGACGTGTGCTCTTCCGATCT**GAGAAGGCCAAGTGGTCCCAGGCC**
EMX_5_target_F	CTCTTTCCCTACACGACGCTCTTCCGATCTNNNN**AAACGGCAGAAGCTGGAGGAGGAAGGG**
EMX_5_target_R	TCAGACGTGTGCTCTTCCGATCT**GGAGGTGACATCGATGTCCTCCCCATTGG**
EMX_14_target_F	CTCTTTCCCTACACGACGCTCTTCCGATCTNNNN**GAAGCAGGCCAATGGGGAGGACATCG**
EMX_14_target_R	TCAGACGTGTGCTCTTCCGATCT**GGAGTGGCCAGAGTCCAGCTTGGG**
GRIN2b_1_target_1_off-target_F	CTCTTTCCCTACACGACGCTCTTCCGATCTNNNN**ATGACAACAAAGATACAGAATACCAGAAGC**
GRIN2b_1_target_1_off-target_R	TCAGACGTGTGCTCTTCCGATCT**GGTGTGATGCTAGGTCTTTCTAACTTTTCC**
GRIN2b_1_target_2_off-target_F	CTCTTTCCCTACACGACGCTCTTCCGATCTNNNN**ACAGAGAGACCCTTTAATTGAAGCCAGG**
GRIN2b_1_target_2_off-target_R	TCAGACGTGTGCTCTTCCGATCT**GAGTGGTGGAAAAGGGGATAGAGTGG**
GRIN2b_1_target_3_off-target_F	CTCTTTCCCTACACGACGCTCTTCCGATCTNNNN**ACACCTCCCATTGTACACACTTGGGAG**
GRIN2b_1_target_3_off-target_R	TCAGACGTGTGCTCTTCCGATCT**GTGCTTTTAACAGGATGAAGTGGATTGGG**
GRIN2b_1_target_4_off-target_F	CTCTTTCCCTACACGACGCTCTTCCGATCTNNNN**TGAAAATAGAGATACCATCTACCAAAATCG**
GRIN2b_1_target_4_off-target_R	TCAGACGTGTGCTCTTCCGATCT**TAGCGATCTTTCTAACTTCTTAATGAAGG**
GRIN2b_1_target_5_off-target_F	CTCTTTCCCTACACGACGCTCTTCCGATCTNNNN**GAACTGAATGAAAATAACAACACAACATAC**
GRIN2b_1_target_5_off-target_R	TCAGACGTGTGCTCTTCCGATCT**GCTTAGGTTATTGATTTGAGACTTTTCTCC**
GRIN2b_1_target_6_off-target_F	CTCTTTCCCTACACGACGCTCTTCCGATCTNNNN**TTTGAAACTAATAAGAACAGACAACATACC**
GRIN2b_1_target_6_off-target_R	TCAGACGTGTGCTCTTCCGATCT**TGAGATCTTTCTAGCTTTCTGATGTGGG**
EMX_1_target_1_off-target_F	CTCTTTCCCTACACGACGCTCTTCCGATCTNNNN**AATCAAAATTTTTCCATGAGGGAGAACAGC**
EMX_1_target_1_off-target_R	TCAGACGTGTGCTCTTCCGATCT**TTGAATTGAGTTCAGGGTGGTGGAAAGG**

EMX_1_target_2_off-target_F	CTCTTTCCCTACACGACGCTCTTCCGATCTNNNN**AGTCAAGATCTGCCCTCAGCCATGTGG**
EMX_1_target_2_off-target_R	TCAGACGTGTGCTCTTCCGATCT**GGATGTCCCAGCTGAAGCACAGAGAGC**
EMX_1_target_3_off-target_F	CTCTTTCCCTACACGACGCTCTTCCGATCTNNNN**ACTGGGGTTCACTTCCTCCTTGTTGCC**
EMX_1_target_3_off-target_R	TCAGACGTGTGCTCTTCCGATCT**GACCTGTGGGTTTTGAAGAAGATGTGGG**
EMX_1_target_4_off-target_F	CTCTTTCCCTACACGACGCTCTTCCGATCTNNNN**TCTGGCCCTGTGTGGGCTTTGATATTGC**
EMX_1_target_4_off-target_R	TCAGACGTGTGCTCTTCCGATCT**TTCAGCTGAGTACTGGTCAGCACACCTG**
EMX_1_target_5_off-target_F	CTCTTTCCCTACACGACGCTCTTCCGATCTNNNN**GCTGGTGAGAGCTTACCTCCACTCAGG**
EMX_1_target_5_off-target_R	TCAGACGTGTGCTCTTCCGATCT**GGATTCTCAGAATGGACTGTCTGAGCTTCC**
EMX_1_target_6_off-target_F	CTCTTTCCCTACACGACGCTCTTCCGATCTNNNN**AGAGGGAAAGAAGGCTGTGTCGGAGCC**
EMX_1_target_6_off-target_R	TCAGACGTGTGCTCTTCCGATCT**CCCCCATCCCACCCCAAGGATGTTCC**
EMX_1_target_7_off-target_F	CTCTTTCCCTACACGACGCTCTTCCGATCTNNNN**GTTATTTATCTCCAAAGAGAAGAGAAAGGG**
EMX_1_target_7_off-target_R	TCAGACGTGTGCTCTTCCGATCT**CCTAGTCTGCCATATATGCTTAAAATGG**
EMX_1_target_8_off-target_F	CTCTTTCCCTACACGACGCTCTTCCGATCTNNNN**ATTACCCAGTCTCTGGTAGTTCTTTATAGC**
EMX_1_target_8_off-target_R	TCAGACGTGTGCTCTTCCGATCT**ATATTTGTCCCTGCCCAAAACTCATGC**
EMX_1_target_9_off-target_F	CTCTTTCCCTACACGACGCTCTTCCGATCTNNNN**CACATTTTAATTTCCGTCTTTTACCTTTCC**
EMX_1_target_9_off-target_R	TCAGACGTGTGCTCTTCCGATCT**GAGGTGTCATTTGATACAGGAATTGACC**

Supplementary Table S6. DNA targets used for assessment of the effect of mismatches in protospacer sequence on PpCas9 DNA cleavage activity *in vitro*.

no substitution	ccttatctcctttcattgagcac**CAACATT**
1	Gcttatctcctttcattgagcac**CAACATT**
3	ccGtatctcctttcattgagcac**CAACATT**
5	ccttCtctcctttcattgagcac**CAACATT**
7	ccttatGtcctttcattgagcac**CAACATT**
9	ccttatctGctttcattgagcac**CAACATT**
11	ccttatctccAttcattgagcac**CAACATT**
13	ccttatctccttAcattgagcac**CAACATT**
15	ccttatctcctttcTttgagcac**CAACATT**
17	ccttatctcctttcatGgagcac**CAACATT**
19	ccttatctcctttcattgCgcac**CAACATT**
21	ccttatctcctttcattgagAac**CAACATT**
23	ccttatctcctttcattgagcaA**CAACATT**

PAM region is shown in bold; substitutions are shown in red.

The PpCas9 amino acid sequence.

MQNNPLNYILGLDLGIASIGWAVVEIDEESSPIRLIDVGVRTFERAEVAKTGESLALSRRL
ARSSRRLIKRRAERLKKAKRLLKAEKILHSIDEKLPINVWQLRVKGLKEKLERQEWAAV
LLHLSKHRGYLSQRKNEGKSDNKELGALLSGIASNHQMLQSSEYRTPAEIAVKKFQVEE
GHIRNQRGSYTHTFSRLDLLAEMELLFQRQAELGNSYTSTTLLENLTALLMWQKPALAG
DAILKMLGKCTFEPSEYKAAKNSYSAERFVWLTKLNNLRILENGTERALNDNERFALLE
QPYEKSKLTYAQVRAMLALSDNAIFKGVRYLGEDKKTVESKTTLIEMKFYHQIRKTLGS
AELKKEWNELKGNSDLLDEIGTAFSLYKTDDDICRYLEGKLPERVLNALLENLNFDKFI
QLSLKALHQILPLMLQGQRYDEAVSAIYGDHYGKKSTETTRLLPTIPADEIRNPVVLRTL
TQARKVINAVVRLYGSPARIHIETAREVGKSYQDRKKLEKQQEDNRKQRESAVKKFKE
MFPHFVGEPKGKDILKMRLYELQQAKCLYSGKSLELHRLLEKGYVEVDHALPFSRTWD
DSFNNKVLVLANENQNKGNLTPYEWLDGKNNSERWQHFVVRVQTSGFSYAKKQRILN
HKLDEKGFIERNLNDTRYVARFLCNFIADNMLLVGKGKRNVFASNGQITALLRHRWGL
QKVREQNDRHHALDAVVVACSTVAMQQKITRFVRYNEGNVFSGERIDRETGEIIPLHFP
SPWAFFKENVEIRIFSENPKLELENRLPDYPQYNHEWVQPLFVSRMPTRKMTGQGHMET
VKSAKRLNEGLSVLKVPLTQLKLSDLERMVNRDREIALYESLKARLEQFGNDPAKAFAE
PFYKKGGALVKAVRLEQTQKSGVLVRDGNGVADNASMVRVDVFTKGGKYFLVPIYTW
QVAKGILPNRAATQGKDENDWDIMDEMATFQFSLCQNDLIKLVTKKKTIFGYFNGLNR
ATSNINIKEHDLDKSKGKLGIYLEVGVKLAISLEKYQVDELGKNIRPCRPTKRQHVR

The DfCas9 amino acid sequence.

MYRFAFDLGTNSLGWAVYRLTNDTRPASIERTGVRIFPNGRDPQSKESNAAGRRMPRG
ARRRQDRSLGRRKRLLDDLIGFGLLPSDADARSAVFAANPIEARARAAREQVALEQLGR
ALWHMSKHRGFKSNRRADKDADEKGKIAIASAALLERLRADGHPTYGAFLHARLVRG
EGTRIRPAGDGAKLSYEFYPTRALLEAEFDHIWAIQAGFHPSLTEAMHERLRDTIFFQRPL
RPVRPGKCTFFPDQDRLPRWHPAAQEFLILSQLNHLRIVDDQGEQPLDITARDLVARTL
MAGTKLSWSGLKKTLKLPSQAEFNLEKGGLKELARNDVAARLLGDTKKPGPLATLWPT
LDSATQEEILWQISEVADPEELICWLMERLGLSREVAERVEKIPLPDGHLRFCKTATQAI
VEKLRCDVIPYDEAVRRAPLLGGAGLDHSDFQAEEGVDTLPPYNRLPVLQRMIGNGTSD
PKDTDLLRYGRITNPTVHIALGQFRRVMNALIAEYGKPAQVVIEATRDMAKSAEELNKI
EKTIRDNEKRNDRWRAELQKAGLLVEGARIGDRFLRMRLWEEIGRGPADRLCPYTGRPI
ALHQLHSDEIEIDHILPFEQTFDDSPANKSLCFRDANRRKGKLSPAAAADRQPDFFDLAAI
IGRTKHLPANKAWRFLPGAMEKWEETRGFEDRQLNATGYLARVVRAYTEALFPKTDA
EGKRRSHVWVLPGRMTAMLRHRWGLNLGDHNRKSRDDHRHHAIDAAVVGVIDRRMV
QVLQTHARNLGVERLDRVLPAPPEPFEGFRDAVLAAVEKVNVSHRAQHGSIDPTDPSQT
SGRLHEDTVFGLVRDVPENQAERTIGNVVVRKPITGLTEKEIGQVRDVKMRLSLQDATS
LARDKSLPEAERKKRLPEALAKWVQETGHRKLRILKPEAGVRPVHDRDGKPYKWLVPG
EISWLDILEAPDGTWFQHATDIWAANSGGAEPWNVAHPSARFIMRVHKNDTIQVFDWD
DRNKCVVEGSNQIKRIVRLEPSANRMRLVGINDAGKFDERHNEPNDPFRWDLATISKLK
LRRARRVRIDELGRVHTIPHGTV
